# Olmesartan alleviates SARS-CoV-2 envelope protein induced renal fibrosis by regulating HMGB1 release and autophagic degradation of TGF-β1

**DOI:** 10.3389/fphar.2023.1187818

**Published:** 2023-05-15

**Authors:** Shilin Zhou, Zanzhe Yu, Zihui Chen, Fengling Ning, Xuetao Hu, Tiangang Wu, Mingxue Li, Hong Xin, Svetlana Reilly, Xuemei Zhang

**Affiliations:** ^1^ Department of Pharmacology, School of Pharmacy, Fudan University, Shanghai, China; ^2^ Department of Nephrology, Renji Hospital, School of Medicine, Shanghai Jiaotong University, Shanghai, China; ^3^ School of Basic Medical Sciences, Fudan University, Shanghai, China; ^4^ Division of Cardiovascular Medicine, Radcliffe Department of Medicine, John Radcliffe Hospital, University of Oxford, Oxford, United Kingdom

**Keywords:** olmesartan, HMGB1, SARS-CoV-2, envelope protein, renal fibrosis, autophagy, TGF-β1

## Abstract

**Background and aims:** Renal damage in severe coronavirus disease 2019 (COVID-19) is highly associated with mortality. Finding relevant therapeutic candidates that can alleviate it is crucial. Angiotensin-converting enzyme inhibitors (ACEIs) and angiotensin-receptor blockers (ARBs) have been shown to be harmless to COVID-19 patients, but it remains elusive whether ACEIs/ARBs have protective benefits to them. We wished to determine if ACEIs/ARBs had a protective effect on the renal damage associated with COVID-19, and to investigate the mechanism.

**Methods:** We used the envelope (E) protein of severe acute respiratory syndrome-coronavirus-2 (SARS-CoV-2) to induce COVID-19-like multiple organ damage and observed renal fibrosis. We induced the epithelial–mesenchymal transformation of HK-2 cells with E protein, and found that olmesartan could alleviate it significantly. The protective effects of olmesartan on E protein-induced renal fibrosis were evaluated by renal-function assessment, pathologic alterations, inflammation, and the TGF-β1/Smad2/3 signaling pathway. The distribution of high-mobility group box (HMGB)1 was examined after stimulation with E protein and olmesartan administration.

**Results:** E protein stimulated HMGB1 release, which triggered the immune response and promoted activation of TGF-β1/Smad2/3 signaling: both could lead to renal fibrosis. Olmesartan regulated the distribution of HMGB1 under E protein stimulation. Olmesartan inhibited the release of HMGB1, and reduced the inflammatory response and activation of TGF-β1/Smad2/3 signaling. Olmesartan increased the cytoplasmic level of HMGB1 to promote the autophagic degradation of TGF-β1, thereby alleviating fibrosis further.

**Conclusion:** Olmesartan alleviates E protein-induced renal fibrosis by regulating the release of HMGB1 and its mediated autophagic degradation of TGF-β1.

## 1 Introduction

Severe acute respiratory syndrome-coronavirus 2 (SARS-CoV-2) has caused a worldwide pandemic and led to coronavirus disease-2019 (COVID-19). In COVID-19, damage to multiple organs caused by SARS-CoV-2 infection hampers treatment and nursing, and increases the mortality risk (Puelles et al., 2020; Xu et al., 2020). Discovering relevant therapeutic candidates that can alleviate such organ damage is a very challenging and unmet need.

Angiotensin-converting enzyme (ACE)2 is the main receptor for SARS-CoV-2 (Hoffmann et al., 2020). ACE2 is also one of the key components of the renin–angiotensin system (RAS) (Danilczyk and Penninger, 2006). Thus, there are valid concerns that use of RAS inhibitors, especially angiotensin-converting enzyme inhibitors (ACEIs) and angiotensin-receptor blockers (ARBs), which are the most commonly used in the clinic, will promote the invasion of SARS-CoV-2 into host cells and aggravate organ damage (Battistoni and Volpe, 2020; Fang et al., 2020). Several studies have investigated the effect of ACEIs/ARBs on SARS-CoV-2 infection and the pathologic process. Some meta-analyses (Zhang P. et al., 2020), single-center/multicenter studies (Zhang X. et al., 2020; Baral et al., 2021; Tetlow et al., 2021) and small-scale clinical trials (Mackey et al., 2022) have shown that use of ACEIs or ARBs does not increase the mortality risk of patients suffering from COVID-19. However, whether ACEIs/ARBs are associated with protective benefits for patients with COVID-19 is unknown.

**FIGURE 1:**
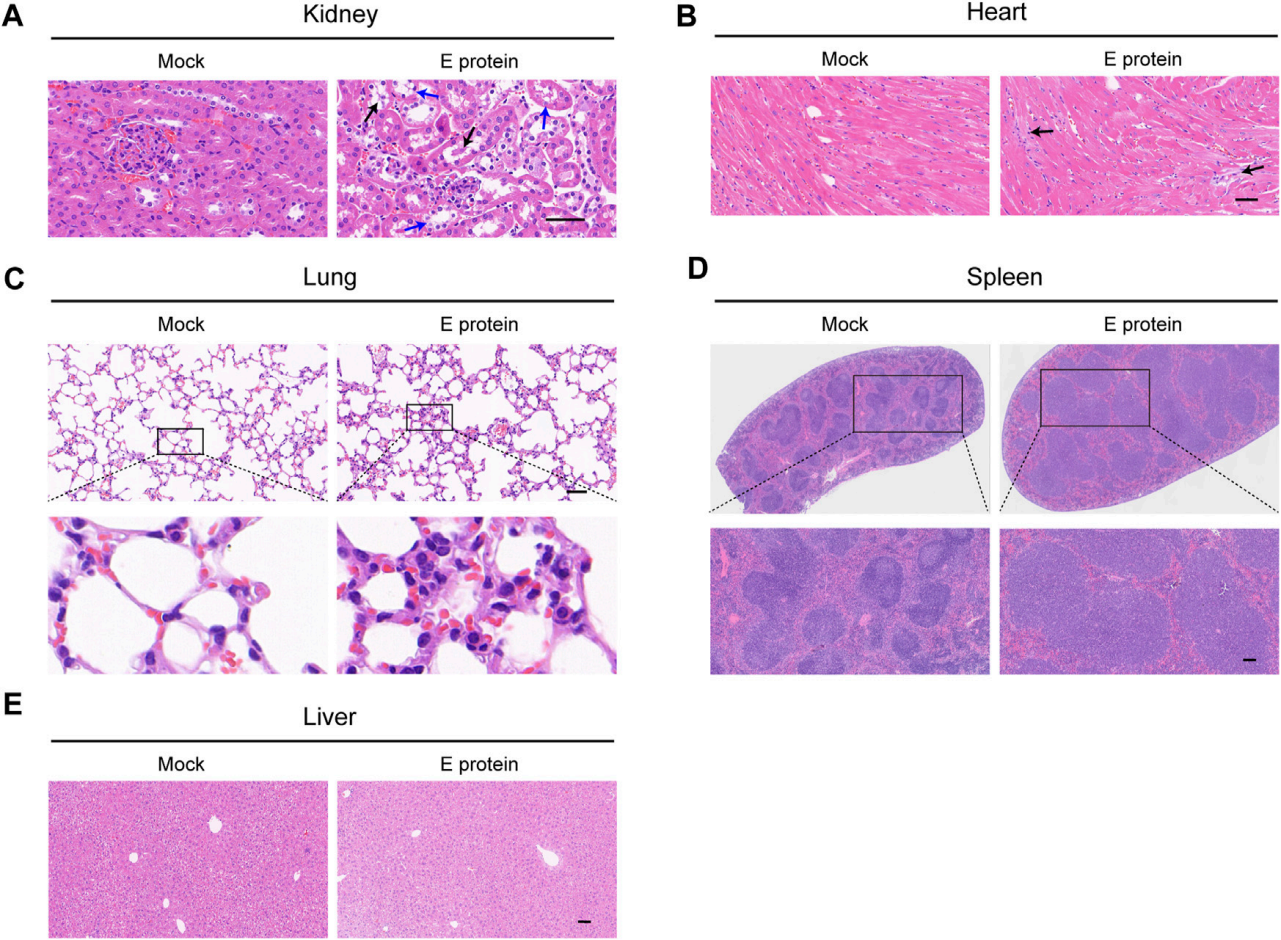
Multiple organ damage induced by E protein. Comparison of histopathological features (H&E staining) of **(A)** kidney (blue arrow: vacuolar degeneration in renal tubular epithelium black arrow: necrosis in renal tubular epithelium), **(B)** heart (black arrow: fibrotic-like structures), **(C)** lung, **(D)** spleen and **(E)** liver. Scale bar = 50 μm.

The envelope (E) protein is one of the main structural proteins of SARS-CoV-2. E protein has been reported to cause inflammation and multi-organ damage as an independent virulence factor (Xia et al., 2021; Zheng et al., 2021). We used the E protein from SARS-CoV-2 to simulate SARS-CoV-2-related organ damage. We found that E protein alone could cause damage to multiple organs, among which renal damage was the most severe (Figure 1). The kidney is one of the main organs damaged by SARS-CoV-2 infection, and renal complications in COVID-19 are associated with an increased risk of death (Pei et al., 2020; Puelles et al., 2020). Various studies have shown that SARS-CoV-2 has renal tropism and can infect the kidney directly to cause renal damage, especially renal-tubule injury and fibrosis (Braun et al., 2020; Puelles et al., 2020; Jansen et al., 2022). Moreover, the kidney is also a key organ of the RAS and an important target of ACEIs/ARBs (Yang and Xu, 2017). Hence, we chose the kidney as a representative organ to study the beneficial effect of ACEIs/ARBs on the organ damage caused by SARS-CoV-2. Then, utilizing an E protein-induced renal-cell model *in vitro*, we screened olmesartan as the most efficacious candidate for a follow-up study (Supplementary Figure S1). Olmesartan belongs to ARBs class of drugs. Like other ARBs, it functions as an angiotensin receptor blocker to undermine the renin-angiotensin-aldosterone system. In clinical practice, olmesartan is mainly used as therapy for hypertension (Kerndt & Soos, 2022).

High-mobility group box (HMGB)1 is a nuclear protein. If released into the extracellular environment, it triggers the immune response and leads to organ damage as damage-associated molecular patterns (DAMPs) (Schaper et al., 2016; Andersson et al., 2018; Zhao et al., 2020). The serum HMGB1 in COVID-19 patients is increased and positively correlated with disease severity (Chen et al., 2020). HMGB1 could be a potential therapeutic target of COVID-19 (Andersson and Tracey, 2011; Bailly and Vergoten, 2020). However, the role of HMGB1 in renal damage associated with COVID-19 is still unclear.

Therefore, we investigated whether olmesartan has a protective effect on the renal damage associated with COVID-19, and demonstrated the role of HMGB1 in it.

## 2 Materials and methods

### 2.1 Reagents

Rabbit anti-α-smooth muscle actin (α-SMA; #ab124964; 1:10,000 dilution) and fibronectin (FN; #ab2413; 1:5,000) antibodies were purchased from Abcam (Cambridge, UK). Rabbit anti-beclin-1 (#3738; 1:1,000), glyceraldehyde 3-phosphate dehydrogenase (GAPDH; #5174; 1:5,000), lamin B1 (#13435; 1:1,000), phosphorylated (p)-beclin-1 (#54101; 1:1,000), p-Smad2/3 (#8828; 1:1,000), Smad23 (#3102; 1:2000), vimentin (#5741; 1:2000) and LC3A/B (#4108; 1:2000) antibodies were purchased from Cell Signaling Technology (Danvers, MA, United States). Rabbit anti-HMGB1 (#A19529; 1:1,000), His tag (#AE086; 1:3,000), P62 (#A19700; 1:1,000) and transforming growth factor (TGF)-β1 (#A16640; 1:1,000) antibodies were purchased from ABclonal Technology (Wuhan, China).

### 2.2 Purification and validation of proteins

Protein purification was carried out as described previously (Xia et al., 2021). In brief, the gene encoding the E protein of SARS-CoV-2 with 6× His tag was cloned in pET28a. Then, the plasmid was transferred into the *Escherichia coli* BL21 (DE3) plysS strain (TransGen Biotech, Beijing, China) to express the full-length E protein. This plasmid was generously provided by Professor Zhaobing Gao (Shanghai Institute of Materia Medica, Chinese Academy of Sciences). The expressed envelope-6×His fusion protein was purified by Ni-NTA resin (Yeasen, Shanghai, China) and eluted with Tris-buffered saline (TBS; Tris-base (20 mM), NaCl (150 mM), pH 8.0) containing imidazole (300 mM). Then, we used the endotoxin removal agarose resin (Yeasen) to remove the endotoxin. The eluent with purified protein was concentrated using centrifugal filter units (Amicon^®^ Ultra, MilliporeSigma, Burlington, MA, United States) and validated by comparison with standard products (Supplementary Figure S2).

### 2.3 Animals and treatment

The protocol for animal experiments was approved by the Animal Care and Use Committee of the School of Pharmacy of Fudan University (Shanghai, China). Twenty-four male C57BL/6 mice (8 weeks) were divided randomly into four groups of six. The mock group was injected with TBS (eluent for purified protein) through the tail vein. The E-protein group was injected with purified E protein (10 mg kg^−1^ body weight) through the tail vein as described previously (Xia et al., 2021). The prophylaxis group was given olmesartan (10 mg kg^−1^ d^−1^ body weight; #HY-17004; MedChemExpress, Monmouth Junction, NJ, United States) by gavage for 4 days before injection with purified E protein. Four days after injection with purified E protein, olmesartan (10 mg kg^−1^ d^−1^ body weight) was administered by gavage to simulate clinical administration. The treatment group was given olmesartan (10 mg kg^−1^ d^−1^ body weight) by gavage 4 days after injection with purified E protein.

On day 32 of the experiment, the mice were anesthetized via inhalation of 5% isoflurane to harvest the blood and various organs as described previously (Wang et al., 2022). Animal studies complied with ARRIVE 2.0 guidelines (Percie Du Sert et al., 2020).

### 2.4 Culture and treatment of cells

RAW 264.7 (mouse leukemia cells of monocyte macrophage, RRID:CVCL_0493) cells were cultured in Dulbecco’s modified Eagle’s medium (Thermo Fisher Scientific, Waltham, MA, United States) containing 10% fetal bovine serum (FBS; Gibco, Grand Island, NY, United States). HK-2 (human kidney proximal tubular epithelial cells, RRID:CVCL_0302) cells were cultured in RPMI 1640 medium (Thermo Fisher Scientific) containing 10% FBS (Gibco) and penicillin/streptomycin (100 units∙mL^−1^; Gibco).

To detect the effect of E protein on renal cells *in vitro*, HK-2 cells (5×10^5^) were seeded in six-well plates with complete medium for 12 h. Then, cells were starved in medium without FBS for 12–16 h. After starvation, cells were stimulated with E protein (ABclonal Technology) at a series of concentrations for 24 h. To detect the efficacy of several representative ACEIs/ARBs, the treatment group also received the corresponding ACEIs/ARBs (20 μM) upon stimulation with 2 μg/mL E protein.

We wished to determine the effect of E protein on macrophages *in vitro* and verify the activity of E protein purified by our research team. RAW 264.7 cells (1×10^6^) were seeded in six-well plates with complete medium for 24 h and stimulated with E protein (ABclonal Technology) or E protein purified by our research team at a final concentration of 1 μg mL^−1^ or 5 μg mL^−1^ for 4 h.

To define the role of HMGB1 in the regulation of autophagy by olmesartan, HK-2 cells were transfected with HMGB1 small interfering (si)RNA (target sequence: CCC​AGA​TGC​TTC​AGT​CAA​CTT; GenePharma, Shanghai, China) by Lipofectamine™ 2000 (Life Technologies, Carlsbad, CA, United States) in Opti-MEM (Gibco). After transfection for 6 h, cells were removed and placed in medium containing 10% FBS for further studies.

### 2.5 Evaluation of renal function

For urine samples, renal function was evaluated by proteinuria, which is quantified by calculating the protein: creatinine ratio in urine (Hada et al., 2022). For blood samples, renal function was evaluated by serum creatinine (SCr) and blood urea nitrogen (BUN) (Wang et al., 2022).

The protein level in urine was measured by a urinary protein test kit. The creatinine level in urine was measured by a creatinine assay kit. The BUN level was measured by a urea assay kit. All kits were obtained from Nanjing Jiancheng Bioengineering Institute (Nanjing, China).

### 2.6 Histology

Organ specimens were fixed in 4% paraformaldehyde for 48 h at room temperature. Then, fixed specimens were embedded in paraffin and sectioned into slices (thickness = 4 μm) for Masson trichrome (kidney specimens only) and hematoxylin and eosin (H&E) staining.

### 2.7 Determination of cytokine concentrations

Cell culture medium and mice serum were harvested for centrifugation (5,000 × *g*. 10 min, 4°C) to remove insoluble materials, and the supernatants were collected. The level of cytokines in supernatants was determined using ELISA kits (Multi Science Biotech, Hangzhou, China) according to manufacturer instructions.

### 2.8 Western blotting

Proteins from kidney tissue or cells were analyzed by Western blotting as described previously (Wang et al., 2022). To determine the total amount of protein, kidney tissue or cells were lysed in RIPA buffer containing 1% phosphatase and a protease inhibitor cocktail (Beyotime Institute of Biotechnology, Shanghai, China) for 30 min on ice.

For fractionation of nuclear/cytosol protein, kidney tissues or cells were treated with nuclear and cytoplasmic extraction reagents (Thermo Fisher Scientific) following manufacturer instructions.

After quantifying the protein content with BCA protein assay kit (Beyotime Institute of Biotechnology), the lysate was added to sodium dodecyl sulfate-polyacrylamide gel electrophoresis (SDS-PAGE) loading buffer (Yeasen) and denatured at 95°C for 10 min. Then, samples underwent SDS-PAGE as described previously (Wang et al., 2022). Densitometry was done using ImageJ (US National Institutes of Health, Bethesda, MD, United States).

### 2.9 Real-time reverse transcription-quantitative polymerase chain reaction (RT-qPCR)

Total RNA was extracted from whole kidney tissue or cells using RNAIso Plus (Takara Biotechnology, Shiga, Japan). Then, complementary-DNA was synthesized using Hifair^®^Ⅱ cDNA Synthesis SuperMix (Yeasen). RT-qPCR was done using TB Green Premix Ex Taq (Takara Biotechnology) following manufacturer instructions. Expression of target genes was normalized to that of GAPDH. The primers for RT-qPCR are listed in Supplementary Table S1.

### 2.10 Ad-GFP-LC3B transfection

HK-2 cells were transfected with ad-GFP-LC3B (HanBio Therapeutics, Shanghai, China) according to manufacturer instructions. Then, they were cultured for 72 h in an atmosphere of 5% CO_2_ at 37°C for subsequent experimental treatment.

### 2.11 Immunofluorescence staining

HK-2 cells were cultured in glass-bottom culture dishes and, after the corresponding treatment, fixed in Immunol Staining Solution (Beyotime Institute of Biotechnology) for 15 min and permeabilized in 0.5% Triton X-100 for 15 min at room temperature. After blockade with Blocking Buffer for Immunol Staining (Beyotime Institute of Biotechnology) for 45 min at room temperature, samples were incubated with a primary antibody at 4°C overnight, and then incubated with secondary Alexa Fluor 594 goat anti-rabbit IgG antibody (1:200; Cell Signaling Technology) for 90 min at room temperature. Nuclear morphology was visualized with 4′,6-diamidino-2-phenylindole (Beyotime Institute of Biotechnology) for 2 min. Images were taken using a confocal microscope (SpinSR10; Olympus, Tokyo, Japan) and processed using FV31S-SW Viewer.

### 2.12 Statistical analyses

Data are the mean ± standard deviation (SD). Results were analyzed using Prism 9.4 (GraphPad, La Jolla, CA, United States). One-way ANOVA was employed to analyze intergroup differences. *p* < 0.05 was considered significant.

## 3 Results

### 3.1 E protein causes severe renal damage

Four weeks after the injection of E protein, several major organs were removed to analyze their histopathological features by H&E staining (Figure 1).

In the kidney, vacuolar degeneration and even necrosis occurred in the epithelial cells of renal tubules. In the spleen, there was obvious edema with a significantly increased number of lymphocytes. In the lung, infiltration of inflammatory cells and thickening of alveolar septa could be observed. In the heart, a few fibrotic-like structures could be observed. In the liver, obvious histopathological changes were not observed.

To summarize, stimulation with E protein at the dose we used caused the most severe damage to the kidney. The renal tubular epithelium was the site that incurred the greatest damage. Therefore, the kidney became the main focus of our study. Renal tubular epithelial cells were employed for mechanistic research.

### 3.2 Olmesartan alleviates the renal damage caused by E protein

**FIGURE 2:**
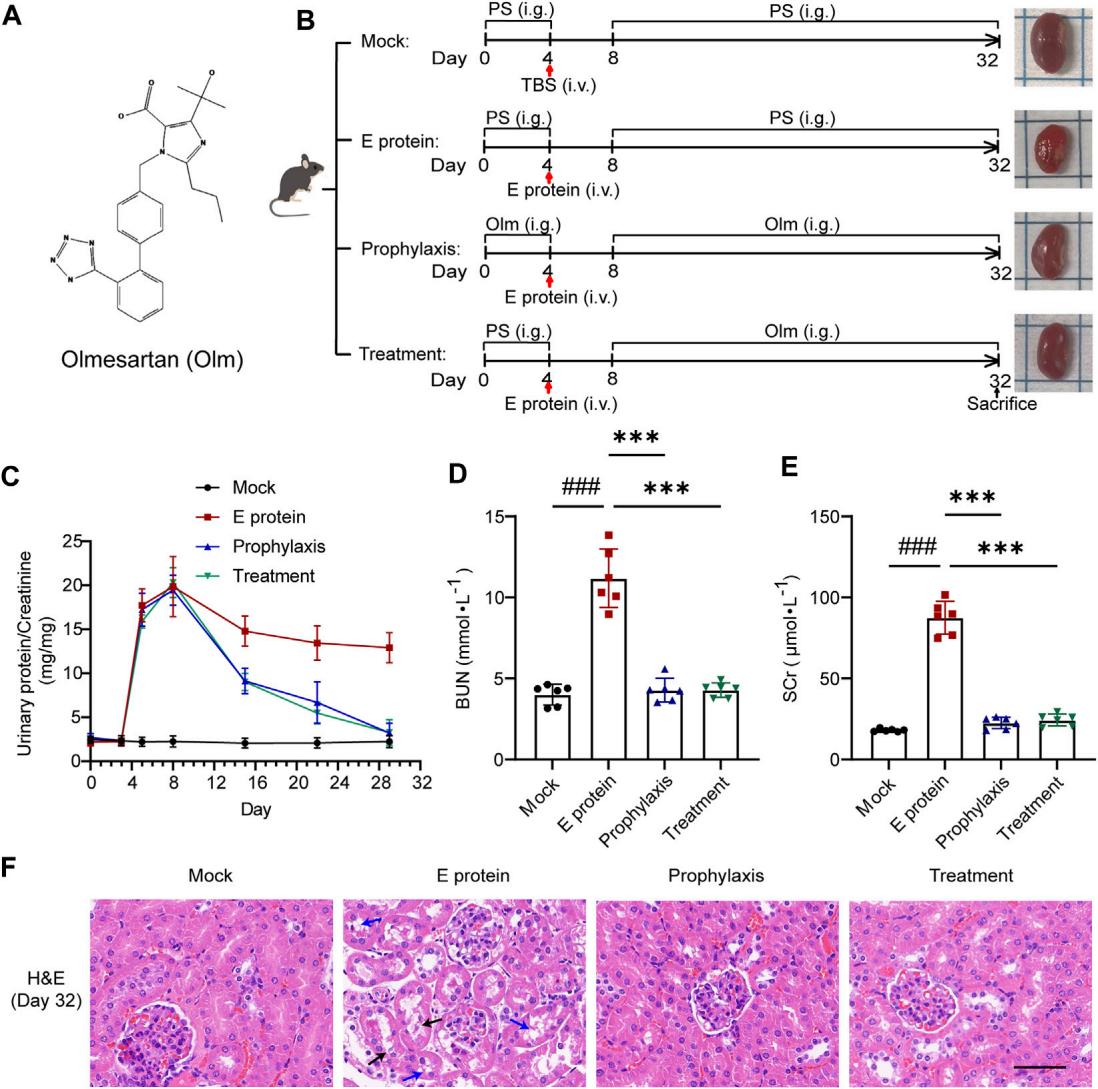
Olmesartan alleviates renal damage caused by E protein. **(A)** Chemical structure of olmesartan (Olm). **(B)** Schematic models show experimental design for animals treatments. **(C–E)** Renal function evaluation (n = 6). Proteinuria was quantified by the ratio of urinary protein to creatinine. On day 32 of the experiment, renal function was evaluated by serum creatinine (SCr) and urea nitrogen (BUN). **(F)** Representative H&E images of kidney samples on day 32 of the experiment (blue arrow: vacuolar degeneration in renal tubular epithelium black arrow: necrosis in renal tubular epithelium scale bar = 50 μm). All the values are means ± SD one-way ANOVA. ###*p* < 0.001 vs. the mock group and *** *p* < 0.001 vs. the E group. PS: physiological saline.

Renal function was evaluated by calculating the ratio of urinary protein: urinary creatinine. E protein could increase the proportion of urinary protein significantly (Figure 2C): renal function was seriously damaged. Prophylactic administration of olmesartan did not alleviate the acute damage of renal function directly, but subsequent therapeutic administration (simulated clinical administration) treated proteinuria and alleviated the damage to renal function (Figures 2C,F; Supplementary Figure S3). These data indicated that olmesartan had a therapeutic role after renal damage rather than a preventive role. Olmesartan also alleviated the damage to other organs caused by E protein (Supplementary Figure S4).

At the end of modeling, we measured the levels of BUN and SCr in the blood of mice in each group (Figures 2D,E). Levels of BUN and SCr in the E-protein group were increased compared with those in the mock group whereas, in the prophylaxis group and treatment group, they were lower compared with those in the E-protein group. These findings were confirmed by H&E staining of kidney tissue (Figure 2F). E protein affected renal function mainly by damaging renal tubular epithelial cells, and olmesartan had a significant protective effect upon renal tubular epithelial cells. Taken together, these results indicated that olmesartan alleviated the renal damage caused by E protein.

### 3.3 Olmesartan improves E protein-induced renal fibrosis

Renal fibrosis is one of the manifestations of end-stage renal disease, but is also a complication of COVID-19. Hence, we detected the mRNA and protein expression of the fibrosis markers: α-SMA, vimentin, and FN in the kidney.

**FIGURE 3:**
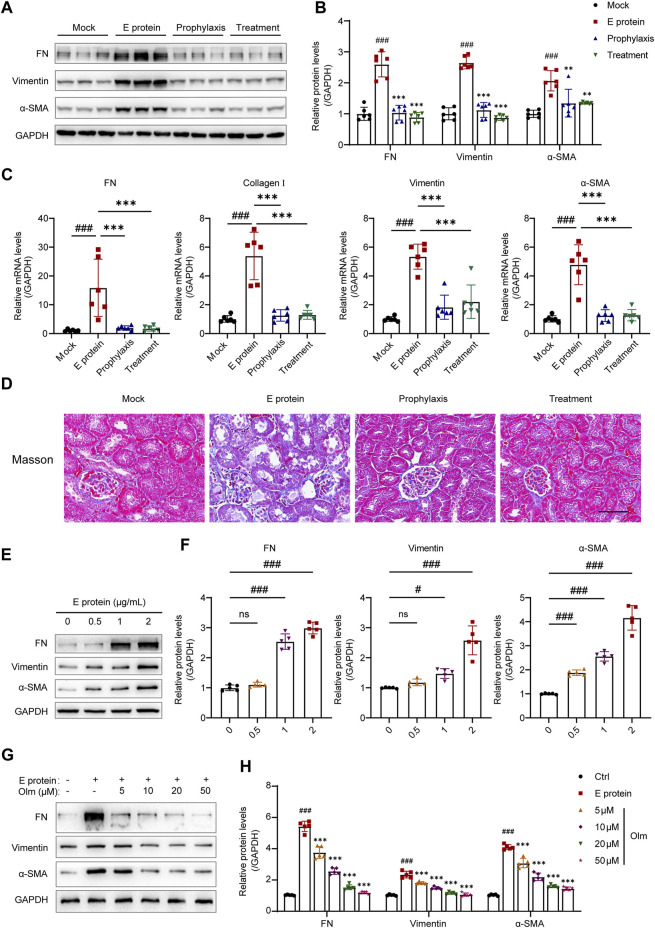
Olmesartan improves E protein-induced renal fibrosis. **(A)** Representative Western blot analysis and **(B)** the relative quantitation of protein expression of fibronectin (FN), vimentin, and α-smooth muscle actin (α-SMA) in the kidneys from each group (n = 6). **(C)** The mRNA expression levels of FN, collagen I, vimentin, and α-SMA in the kidneys from each group (n = 6). **(D)** Representative Masson trichrome images of kidney samples (scale bar = 50 μm). **(E, F)** E protein induced the epithelial mesenchymal transformation (EMT) of HK-2 cells in a dose-dependent manner (n = 5). **(G, H)** Olmesartan inhibited the epithelial mesenchymal transformation (EMT) of HK-2 cells induced by E protein in a dose-dependent manner (n = 5). All the values are means ± SD one-way ANOVA. ns *p* > 0.05, #*p* < 0.05, ###*p* < 0.001 vs. the mock or control group and *** *p* < 0.001 vs. the E protein group.

Treatment with E protein upregulated the mRNA and protein expression of fibrosis markers (Figures 3A–C), which indicated that renal fibrosis had occurred. In the prophylaxis group and treatment group, the level of fibrosis markers was decreased significantly, but there was no significant difference between these two groups, which was consistent with the results shown in Figure 2. Masson trichrome staining also revealed consistent results (Figure 3D). Collagen deposition in the E-protein group was obvious, whereas that in the prophylaxis group and treatment group was alleviated. We also detected activation of the TGF-β1/Smad2/3 signaling pathway stimulated by E protein, which was inhibited significantly by olmesartan (Supplementary Figure S5). Taken together, these data suggested that E protein can induce renal fibrosis *in vivo*, whereas olmesartan could alleviate it effectively.

*In vitro*, we stimulated HK-2 cells with E protein, and found that E protein could stimulate HK-2 to undergo epithelial–mesenchymal transformation (EMT) in a dose-dependent manner (Figures 3E,F). Olmesartan could inhibit EMT *in vitro* in a dose-dependent manner (Figures 3G,H).

To summarize, these results demonstrated that olmesartan improved E protein-induced renal fibrosis *in vivo* and *in vitro*.

### 3.4 Olmesartan alleviates inflammation by inhibiting HMGB1 release

E-protein stimulation could induce an inflammatory response in multiple organs (Figure 1). Long-term inflammation often leads to organ damage. HMGB1 can trigger an inflammatory response and subsequent organ damage as DAMPs only after it has been released into the extracellular environment (Schaper et al., 2016; Andersson et al., 2018; Zhao et al., 2020). Therefore, we tested the effect of E-protein stimulation and olmesartan treatment on HMGB1 release.

**FIGURE 4:**
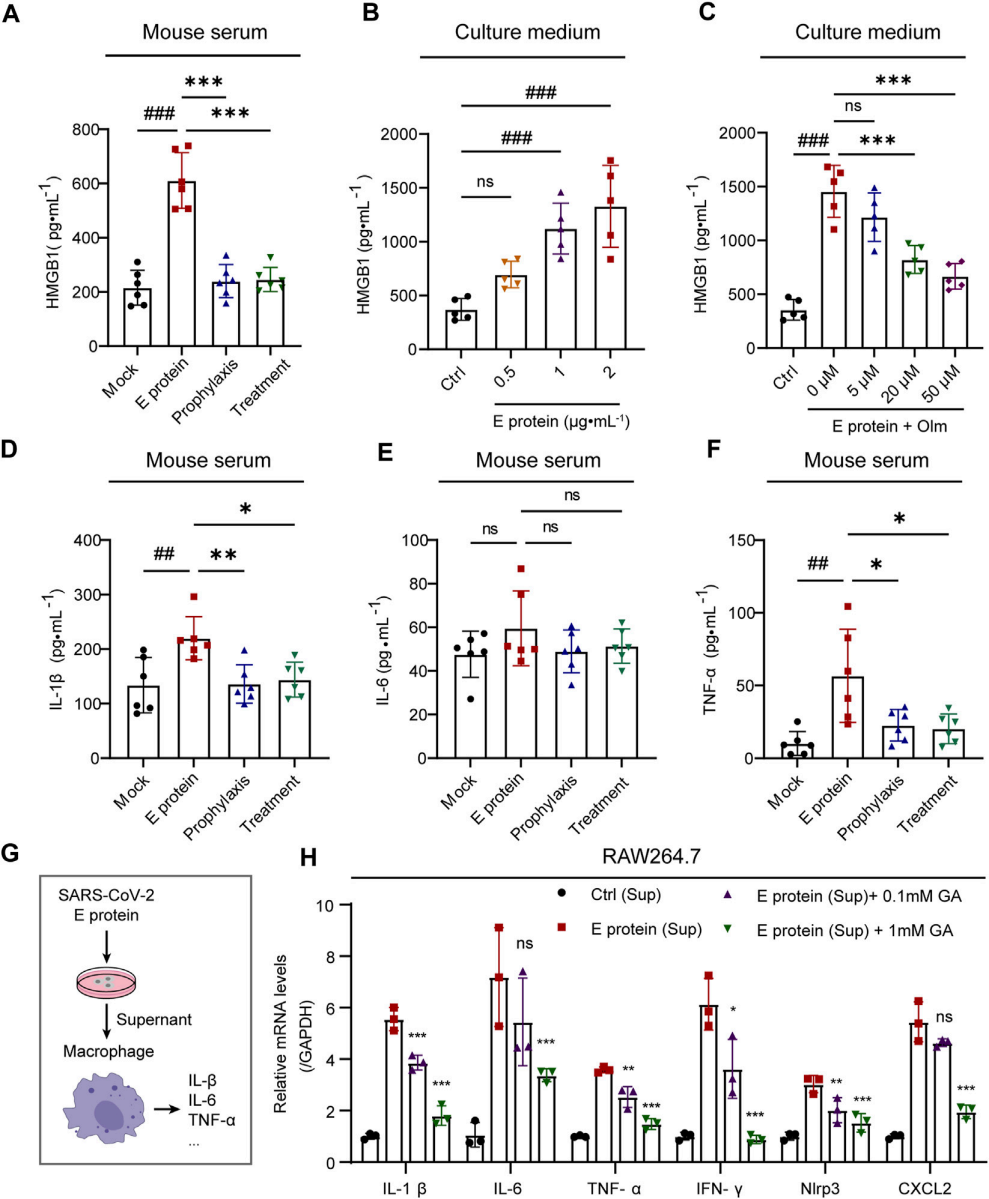
Olmesartan alleviates inflammation by inhibiting HMGB1 release. **(A)** The level of released HMGB1 in mouse serum (n = 6). **(B)** The level of released HMGB1 in HK-2 cell culture medium after 24 h of stimulation with a series of concentrations of E protein (n = 5). **(C)** In the presence of 2 μg mL^-1^ E protein, the level of HMGB1 released in HK-2 cell culture medium after 24 h of treatment with a series of concentrations of olmesartan (n = 5). The level of **(D)** IL-1β, **(E)** IL-6 and **(F)** TNF-α in mouse serum (n = 6). **(G)** Schematic diagram of experimental treatment for **(H)**. **(H)** mRNA expression of inflammatory cytokines in RAW264.7 cells (n = 3). E protein (Sup) group: after the HK-2 cells were stimulated by E protein for 24h, the culture medium supernatant was collected and treated with RAW264.7 cells for 2 h with or without Glycyrrhizic acid. Ctrl group: E protein was added to in cell-free culture medium and simulated to cultured for 24 h, the supernatant of the medium was collected and treated with RAW264.7 cells for 2 h. All the values are means ± SD one-way ANOVA. ns *p* > 0.05, ##*p* < 0.01, ###*p* < 0.001 vs. the mock or control group and * *p* < 0.05, ** *p* < 0.01, *** *p* < 0.001 vs. the E protein or E protein (Sup) group. Sup: culture medium supernatant. GA: Glycyrrhizic acid, inhibitor of HMGB1.

Stimulation with E protein promoted the release of HMGB1 significantly, whereas olmesartan inhibited its release and reduced the level of proinflammatory cytokines in serum (Figures 4A, D–F). Moreover, E protein could stimulate HMGB1 release in a dose-dependent fashion. The contrary effect of olmesartan was also dose-dependent (Figures 4B,C). These results indicated that HMGB1 was probably a key factor in the inflammation caused by E protein, and also the key factor in the therapeutic role of olmesartan.

To further demonstrated the role of released HMGB1 in inflammation, we stimulated HK-2 cells with E protein and gathered the supernatant of the culture medium. Then, we treated mouse macrophages (RAW 264.7 cells) with the supernatant. The supernatant could stimulate expression of the proinflammatory cytokines of macrophages compared with that in the control group. Importantly, glycyrrhizic acid (HMGB1 inhibitor) could inhibit expression of proinflammatory cytokines in a dose-dependent manner (Figure 4H).

To summarize, olmesartan alleviated inflammation by inhibiting HMGB1 release.

### 3.5 Olmesartan promotes the autophagic degradation of TGF-β1 by increasing the cytoplasmic level of HMGB1

**FIGURE 5:**
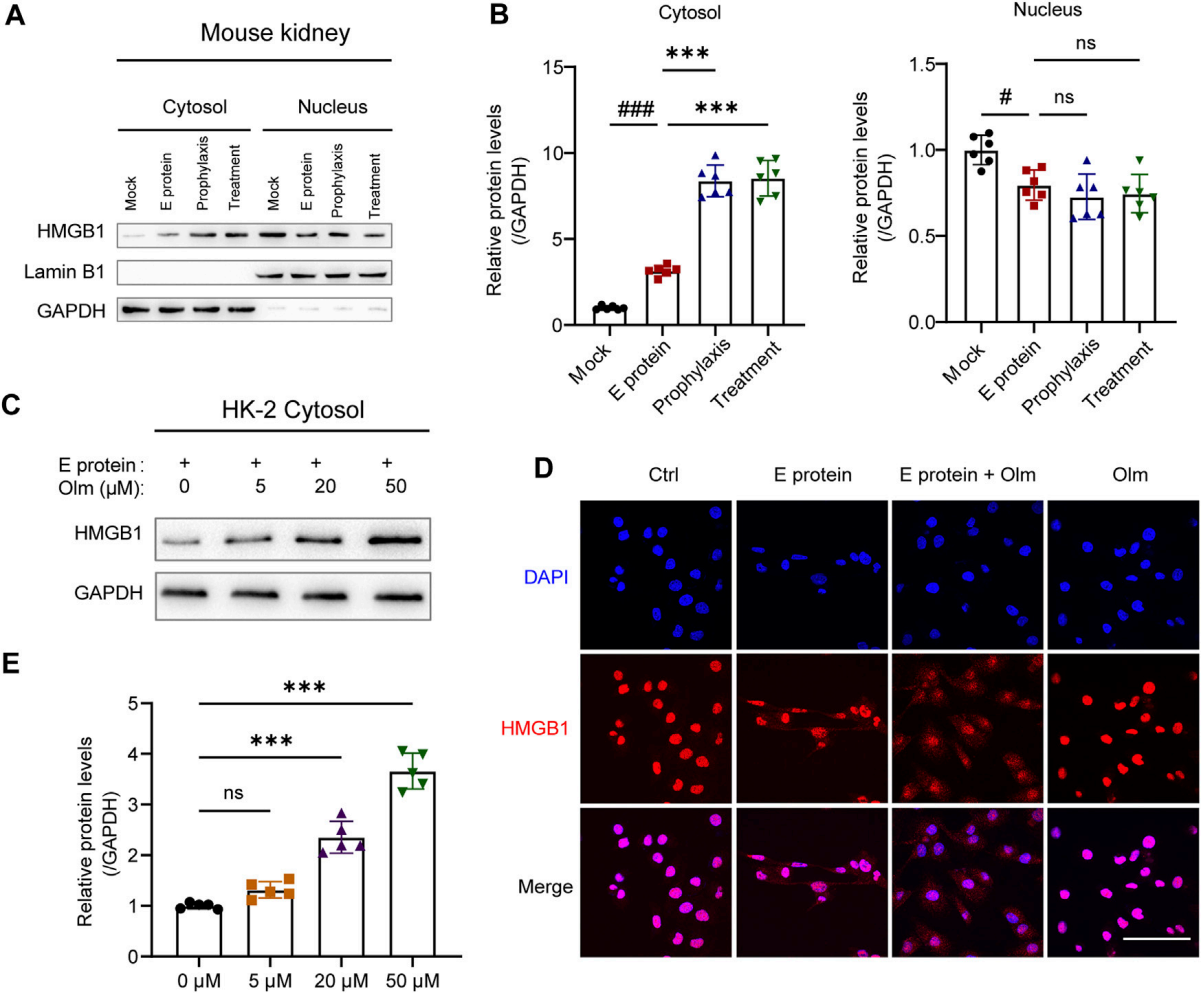
Olmesartan upregulates the cytoplasmic level of HMGB1. **(A)** The distribution of HMGB1 in nucleus and cytoplasm of kidneys and **(B)** the relative quantitation. **(C)** In the presence of 2 μg mL^-1^ E protein, the protein level of HMGB1 in the cytoplasm of HK-2 cells after 24 h of treatment with a series of concentrations of olmesartan (n = 5). **(D)** Representative fluorescent images of HMGB1 distribution under different treatment (scale bar = 100 μm). **(E)** The relative quantitation of **(C)**. All the values are means ± SD one-way ANOVA. ns *p* > 0.05 #*p* < 0.05, ###*p* < 0.001 vs. the mock group and *** *p* < 0.001 vs. the E protein group.

HMGB1 release was inhibited by olmesartan, and its level in the cytoplasm increased accordingly (Figures 5A,B, Supplementary Figure S6). Furthermore, under E-protein stimulation, the upregulation of cytoplasmic HMGB1 expression by olmesartan was dose-dependent (Figures 5C,E). The results of immunofluorescence staining confirmed this phenomenon (Figure 5D). Under stimulation by E protein, olmesartan increased the distribution of HMGB1 in the cytoplasm significantly. Interestingly, use of olmesartan alone did not affect the distribution of HMGB1. Hence, olmesartan only inhibited the release of HMGB1 stimulated by E protein, and did not promote its translocation from the nucleus to the cytoplasm.

**FIGURE 6:**
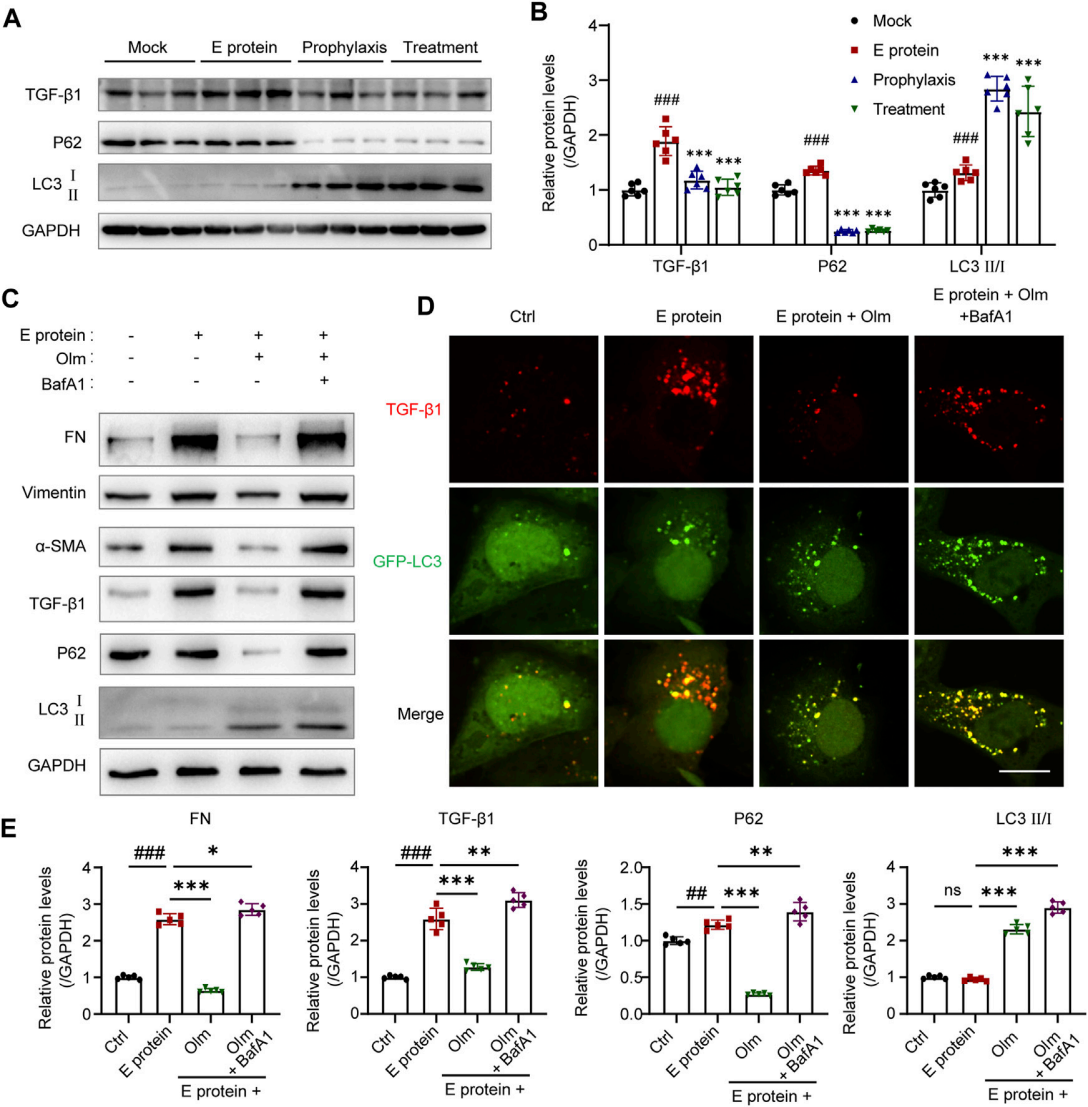
Olmesartan promotes autophagic degradation of TGF-β1 in renal tubular epithelial cells. **(A)** Autophagic degradation of TGF-β1 in the kidneys from each group (n = 6) and **(B)** the relative quantitation. **(C)** The level of EMT and autophagy-related proteins in HK-2 cells (n = 5) and **(E)** the relative quantitation. **(D)** Expression and distribution of GFP-LC3B and TGF-β1 was detected. HK-2 cells were transfected with ad-GFP-LC3B in different treatment. Representative immunofluorescence images were shown (scale bar = 20 μm). All the values are means ± SD one-way ANOVA. ns *p* > 0.05, ##*p* < 0.01, ###*p* < 0.001 vs. the control group and * *p* < 0.05, ** *p* < 0.01, *** *p* < 0.001 vs. the E protein group. BafA1: Bafilomycin A1, autophagy degradation inhibitor.

Cytoplasmic HMGB1 can activate autophagy by interacting with beclin-1 (Tang et al., 2010; Cadwell, 2016). Ding and colleagues showed that TGF-β1 (key factor in fibrosis) was degraded by autophagy in the cytoplasm (Ding et al., 2014): our data validated their result (Supplementary Figure S7). Thus, we detected the activation of autophagy and TGF-β1 level in the kidney. Olmesartan activated autophagy significantly and downregulated protein expression of intracellular TGF-β1 (Figures 6A,B). To further investigate if olmesartan downregulated TGF-β1 expression by activating autophagy, we used BafA1 (inhibitor of autophagy degradation) to interfere with the effect of olmesartan. The presence of BafA1 increased the expression of TGF-β1 and fibrosis-related proteins significantly (Figures 6C,E). Immunofluorescence staining also showed that BafA1 increased the relative fluorescence intensity of intracellular TGF-β1 significantly, and that TGF-β1 and GFP-LC3 were strongly co-located (Figure 6D). These data indicated that olmesartan promoted the autophagic degradation of TGF-β1.

**FIGURE 7:**
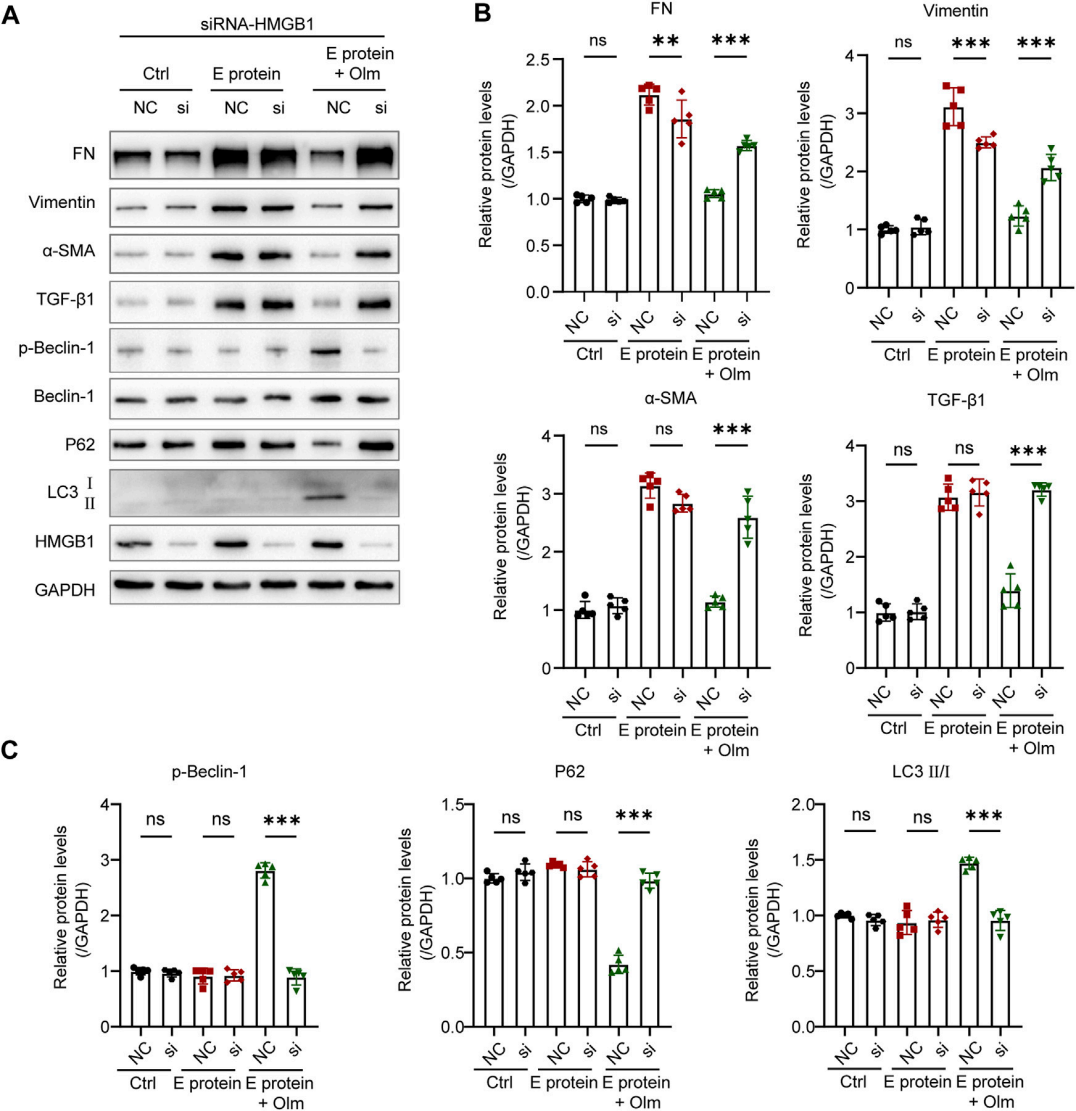
The regulation of Olmesartan on autophagy is mediated by HMGB1. **(A)** Effect of interfering HMGB1 expression on olmesartan regulating EMT and autophagy-related proteins and **(B, C)** the relative quantitation (n = 5). All the values are means ± SD one-way ANOVA. ns *p* > 0.05 ** *p* < 0.01, *** *p* < 0.001 vs. the E protein + Olm (NC) group. NC: siRNA for negative control si: siRNA targeting HMGB1.

Olmesartan promoted the phosphorylation of beclin-1 (Figure 7), which is a vital step in autophagy. This phenomenon occurred because increased numbers of cytoplasmic HMGB1 molecules interacted with beclin-1 and promoted its phosphorylation (Tang et al., 2010; Xue et al., 2020). To further confirm the key role of HMGB1 in the regulation of autophagy by olmesartan, we used siRNA targeting HMGB1 to interfere with HMGB1 expression in HK-2 cells. Then, the cells continued to be treated with E protein and/or olmesartan. As shown in Figure 7, after interfering with HMGB1 expression, the regulation autophagy by olmesartan was diminished. Meanwhile, the effect of olmesartan on downregulating expression of TGF-β1 and fibrosis markers was also diminished. These results revealed that HMGB1 had an indispensable role in the regulation of autophagy by olmesartan.

Taken together, these results indicated that olmesartan could promote the autophagic degradation of TGF-β1 by increasing HMGB1 level in the cytoplasm.

## 4 Discussion

Using a model of E protein-induced renal damage in mice, we provided convincing evidence for using olmesartan to treat patients with renal fibrosis associated with COVID-19. E protein stimulates the release of HMGB1, which can promote activation of the TGF-β1/Smad2/3 signaling pathway (Zou et al., 2021) and trigger the inflammatory response (Li et al., 2021), both of which contribute to renal fibrosis. Notably, olmesartan could inhibit the release of HMGB1 induced by E protein, thereby reducing the inflammatory response and activation of TGF-β1/Smad2/3 signaling. Olmesartan could also increase the HMGB1 level in cytoplasm to promote the autophagic degradation of TGF-β1, which further alleviates fibrosis. Taken together, our findings provide a strong support for the hypothesis that ACEIs/ARBs are beneficial for COVID-19 patients.

Xia and colleagues showed that stimulation with E protein alone could cause acute respiratory distress syndrome-like damage *in vitro* and *in vivo*. The main damage detected in their study was pulmonary congestion and spleen edema, which was due to an excessive inflammatory response (Xia et al., 2021). We detected the pathologic damage to several major organs induced by E protein. The kidney was the most severely damaged organ, and was characterized by necrosis of renal tubular epithelial cells, renal interstitial inflammation, and fibrosis. The reason for this difference is the modeling dosage and time. Xia and colleagues used a high dose of E protein (25 mg kg^−1^ body weight) and observed outcomes after 4 days. We used a low dose of E protein (10 mg kg^−1^ body weight) and continued observation for 28 days before collecting various organs for analyses. High-dose administration focuses on acute injury, so the long-term effects of E protein cannot be observed. We focused more on the long-term impact of SARS-CoV-2 infection on human health and the role of E protein in it, which could be meaningful for COVID-19 treatment. Furthermore, the renal damage induced by E protein is similar to that of COVID-19 detected clinically (Han and Ye, 2021; Nie et al., 2021; Jansen et al., 2022), and the renal complications in COVID-19 are associated with higher risk of mortality (Pei et al., 2020; Han and Ye, 2021). Our data revealed the key role of E protein in the pathology of SARS-CoV-2 infection and the importance of kidney studies for COVID-19.

The most significant findings of our study were the discovery and validation of the regulatory effect of olmesartan on HMGB1 under stress (E-protein stimulation). HMGB1 is a chromatin-associated nuclear protein. It can translocate from the nucleus to the cytoplasm under certain types of stress, and then be secreted into the extracellular space in active or passive manners (Volchuk et al., 2020; Kim et al., 2021). Nuclear HMGB1 regulates the repair of DNA damage and genome stability as a non-histone, chromatin-associated protein and DNA “chaperone” (Wang and Zhang, 2020). Cytoplasmic HMGB1 can interact with beclin-1 and activate its phosphorylation to induce autophagy (Tang et al., 2010; Xue et al., 2020). HMGB1 released into the extracellular environment is a risk factor for triggering the immune response (Schaper et al., 2016; Andersson et al., 2018; Zhao et al., 2020). HMGB1 is found in high concentrations in the serum of patients with severe COVID-19 (Chen et al., 2020). The same change is also observed in chronic kidney disease (CKD). (Leelahavanichkul et al., 2011). Released HMGB1 can ultimately lead to renal fibrosis by triggering inflammation (Li et al., 2021) and activating TGF-β1/Smad2/3 signaling (Zou et al., 2021). Therefore, HMGB1 is considered to be a potential therapeutic target for COVID-19 and CKD (Andersson and Tracey, 2011; Bailly and Vergoten, 2020; Zhao et al., 2020).

The protective effect of ARBs on the kidney is well known, but studies on how ARBs regulate HMGB1 are still blank. We found, for the first time, that olmesartan can regulate the distribution of HMGB1 under the stimulation of E protein. Olmesartan can inhibit the release of HMGB1 under stress significantly, thus inhibiting the excessive immune response and long-term inflammation, which promotes the development of CKD and ultimately leads to renal fibrosis. Furthermore, with the inhibition of HMGB1 release, olmesartan increased the level of HMGB1 in the cytoplasm significantly, thus activating autophagy. Notably, HMGB1-mediated autophagy promoted the autophagic degradation of TGF-β1, which inhibits the secretion of TGF-β1 and activation of TGF-β1/Smad2/3 signaling, thereby alleviating fibrosis further. These effects were greatly diminished after interfering with HMGB1 expression. These findings indicate that HMGB1 is an indispensable factor in olmesartan’s remission of renal damage induced by E protein.

Olmesartan is a traditional antihypertensive drug, so we also monitored the blood pressure, heart rate, and body weight of mice during experiments (Supplementary Figure S8). E-protein stimulation could reduce the blood pressure and heart rate of mice temporarily, reduce the vitality of mice significantly, and lead to body weight loss. After 4 days, the heart rate and blood pressure of mice in the E-protein group increased gradually and were not different from those in the mock group. However, their body weight was significantly lower than that in the mock group, which further displayed the damage of E protein caused to organs. Olmesartan can restore the weight of mice, and has no effect on heart rate. At the experimental dose, olmesartan kept the blood pressure of mice low, but no adverse events were observed in heart, liver, spleen and lung (Supplementary Figure S4). This is consistent with clinical practice. Olmesartan has been known for its relatively mild side effect. In clinical practice, the most common side effect of olmesartan is headache, which up to 7% of patients may experience, and 3% may have associated with dizziness. And side effects on other organs are relatively rare (Kerndt & Soos, 2022).

## 5 Conclusion

We provided the first evidence that olmesartan regulates the distribution of HMGB1 under stress. Stimulation using E protein could trigger HMGB1 release, induce renal damage and development into CKD and, finally, lead to renal fibrosis. This scenario is similar to the pathologic process of renal fibrosis associated with COVID-19. Olmesartan inhibited the release of HMGB1, thereby blunting inflammation. With the inhibition of HMGB1 release, olmesartan increased the level of HMGB1 in the cytoplasm, thus mediating the autophagic degradation of TGF-β1 and inhibiting renal fibrosis further. Olmesartan is a potential candidate for preventing the development of renal fibrosis associated with COVID-19, and HMGB1 is an important therapeutic target.

## Data Availability

The original contributions presented in the study are included in the article/Supplementary Material, further inquiries can be directed to the corresponding authors.

## Abbreviations

ACE2, angiotensin-converting enzyme 2; ACEI, angiotensin-converting enzyme inhibitor; ARB, angiotensin-receptor blocker; BafA1, bafilomycin A1; BUN, blood urea nitrogen; CKD, chronic kidney disease; COVID-19, coronavirus disease-2019; DAMP, damage-associated molecular pattern; E, envelope; EMT, epithelial–mesenchymal transformation; FN, fibronectin; GA, glycyrrhizic acid; HMGB1, high mobility group box 1; Olm, olmesartan; PS, physiological saline; RAS, renin–angiotensin system; SARS-CoV-2, severe acute respiratory syndrome coronavirus 2; SCr, serum creatinine; SMA, smooth muscle actin; Sup, culture medium supernatant; TGF-β1, transforming growth factor beta-1.

## References

[B1] Andersson U. Tracey K. J. (2011). HMGB1 is a therapeutic target for sterile inflammation and infection. Annu. Rev. Immunol. 29, 139–162. 10.1146/annurev-immunol-030409-101323 21219181 PMC4536551

[B2] Andersson U. Yang H. Harris H. (2018). High-mobility group box 1 protein (HMGB1) operates as an alarmin outside as well as inside cells. Semin. Immunol. 38, 40–48. 10.1016/j.smim.2018.02.011 29530410

[B3] Bailly C. Vergoten G. (2020). Glycyrrhizin: An alternative drug for the treatment of COVID-19 infection and the associated respiratory syndrome? Pharmacol. Ther. 214, 107618. 10.1016/j.pharmthera.2020.107618 32592716 PMC7311916

[B4] Baral R. Tsampasian V. Debski M. Moran B. Garg P. Clark A. (2021). Association between renin-angiotensin-aldosterone system inhibitors and clinical outcomes in patients with COVID-19: A systematic review and meta-analysis. JAMA Netw. Open 4, e213594. 10.1001/jamanetworkopen.2021.3594 33787911 PMC8013817

[B5] Battistoni A. Volpe M. (2020). Might renin-angiotensin system blockers play a role in the COVID-19 pandemic? Eur. Heart. J. Cardiovasc. Pharmacother. 6, 248–251. 10.1093/ehjcvp/pvaa030 32286607 PMC7184353

[B6] Braun F. Lütgehetmann M. Pfefferle S. Wong M. N. Carsten A. Lindenmeyer M. T. (2020). SARS-CoV-2 renal tropism associates with acute kidney injury. Lancet 396, 597–598. 10.1016/S0140-6736(20)31759-1 32818439 PMC7431179

[B7] Cadwell K. (2016). Crosstalk between autophagy and inflammatory signalling pathways: Balancing defence and homeostasis. Nat. Rev. Immunol. 16, 661–675. 10.1038/nri.2016.100 27694913 PMC5343289

[B8] Chen R. Huang Y. Quan J. Liu J. Wang H. Billiar T. R. (2020). HMGB1 as a potential biomarker and therapeutic target for severe COVID-19. Heliyon 6, e05672. 10.1016/j.heliyon.2020.e05672 33313438 PMC7720697

[B9] Danilczyk U. Penninger J. M. (2006). Angiotensin-converting enzyme II in the heart and the kidney. Circ. Res. 98, 463–471. 10.1161/01.RES.0000205761.22353.5f 16514079

[B10] Ding Y. Kim S. L. Lee S. Koo J. K. Wang Z. Choi M. E. (2014). Autophagy regulates TGF-β expression and suppresses kidney fibrosis induced by unilateral ureteral obstruction. J. Am. Soc. Nephrol. 25, 2835–2846. 10.1681/ASN.2013101068 24854279 PMC4243349

[B11] Fang L. Karakiulakis G. Roth M. (2020). Are patients with hypertension and diabetes mellitus at increased risk for COVID-19 infection? Lancet Respir. Med. 8, e21. 10.1016/S2213-2600(20)30116-8 32171062 PMC7118626

[B12] Hada I. Shimizu A. Takematsu H. Nishibori Y. Kimura T. Fukutomi T. (2022). A novel mouse model of idiopathic nephrotic syndrome induced by immunization with the podocyte protein Crb2. J. Am. Soc. Nephrol. 33, 2008–2025. 10.1681/ASN.2022010070 35985815 PMC9678040

[B13] Han X. Ye Q. (2021). Kidney involvement in COVID-19 and its treatments. J. Med. Virol. 93, 1387–1395. 10.1002/jmv.26653 33150973

[B14] Hoffmann M. Kleine-Weber H. Schroeder S. Krüger N. Herrler T. Erichsen S. (2020). SARS-CoV-2 cell entry depends on ACE2 and TMPRSS2 and is blocked by a clinically proven protease inhibitor. Cell 181, 271–280. 10.1016/j.cell.2020.02.052 32142651 PMC7102627

[B15] Jansen J. Reimer K. C. Nagai J. S. Varghese F. S. Overheul G. J. de Beer M. (2022). SARS-CoV-2 infects the human kidney and drives fibrosis in kidney organoids. Cell Stem Cell 29, 217–231.e8. 10.1016/j.stem.2021.12.010 35032430 PMC8709832

[B16] Kerndt C. C. Soos M. P. (2022). “Olmesartan,” in StatPearls (StatPearls Publishing). 31335087

[B17] Kim Y. H. Kwak M. S. Lee B. Shin J. M. Aum S. Park I. H. (2021). Secretory autophagy machinery and vesicular trafficking are involved in HMGB1 secretion. Autophagy 17, 2345–2362. 10.1080/15548627.2020.1826690 33017561 PMC8496717

[B18] Leelahavanichkul A. Huang Y. Hu X. Zhou H. Tsuji T. Chen R. (2011). Chronic kidney disease worsens sepsis and sepsis-induced acute kidney injury by releasing high mobility group box protein-1. Kidney Int. 80, 1198–1211. 10.1038/ki.2011.261 21832986 PMC3491658

[B19] Li Y. Yuan Y. Huang Z. Chen H. Lan R. Wang Z. (2021). GSDME-mediated pyroptosis promotes inflammation and fibrosis in obstructive nephropathy. Cell Death Differ. 28, 2333–2350. 10.1038/s41418-021-00755-6 33664482 PMC8329275

[B20] Mackey K. Kansagara D. Vela K. (2022). Update alert 9: Risks and impact of angiotensin-converting enzyme inhibitors or angiotensin-receptor blockers on SARS-CoV-2 infection in adults. Ann. Intern. Med. 175, W47–W48. 10.7326/L21-0791 35130048 PMC8855787

[B21] Nie X. Qian L. Sun R. Huang B. Dong X. Xiao Q. (2021). Multi-organ proteomic landscape of COVID-19 autopsies. Cell 184, 775–791.e14. 10.1016/j.cell.2021.01.004 33503446 PMC7794601

[B22] Pei G. Zhang Z. Peng J. Liu L. Zhang C. Yu C. (2020). Renal involvement and early prognosis in patients with COVID-19 pneumonia. J. Am. Soc. Nephrol. 31, 1157–1165. 10.1681/ASN.2020030276 32345702 PMC7269350

[B23] Percie du Sert N. Hurst V. Ahluwalia A. Alam S. Avey M. T. Baker M. (2020). The ARRIVE guidelines 2.0: Updated guidelines for reporting animal research. Plos Biol. 18, 3793–3801. 10.1113/JP280389 PMC7610696 32666574

[B24] Puelles V. G. Lütgehetmann M. Lindenmeyer M. T. Sperhake J. P. Wong M. N. Allweiss L. (2020). Multiorgan and renal tropism of SARS-CoV-2. N. Engl. J. Med. 383, 590–592. 10.1056/NEJMc2011400 32402155 PMC7240771

[B25] Schaper F. de Leeuw K. Horst G. Bootsma H. Limburg P. C. Heeringa P. (2016). High mobility group box 1 skews macrophage polarization and negatively influences phagocytosis of apoptotic cells. Rheumatol. Oxf. 55, 2260–2270. 10.1093/rheumatology/kew324 27632996

[B26] Tang D. Kang R. Livesey K. M. Cheh C. Farkas A. Loughran P. (2010). Endogenous HMGB1 regulates autophagy. J. Cell Biol. 190, 881–892. 10.1083/jcb.200911078 20819940 PMC2935581

[B27] Tetlow S. Segiet-Swiecicka A. O'Sullivan R. O'Halloran S. Kalb K. Brathwaite-Shirley C. (2021). ACE inhibitors, angiotensin receptor blockers and endothelial injury in COVID-19. J. Intern. Med. 289, 688–699. 10.1111/joim.13202 33210357 PMC7753609

[B28] Volchuk A. Ye A. Chi L. Steinberg B. E. Goldenberg N. M. (2020). Indirect regulation of HMGB1 release by gasdermin D. D. Nat. Commun. 11, 4561. 10.1038/s41467-020-18443-3 32917873 PMC7486936

[B29] Wang S. Zhang Y. (2020). HMGB1 in inflammation and cancer. J. Hematol. Oncol. 13 (1), 116. 10.1186/s13045-020-00950-x 32831115 PMC7443612

[B30] Wang M. Zeng F. Ning F. Wang Y. Zhou S. He J. (2022). Ceria nanoparticles ameliorate renal fibrosis by modulating the balance between oxidative phosphorylation and aerobic glycolysis. J. Nanobiotechnol. 20, 3. 10.1186/s12951-021-01122-w PMC8725394 34983531

[B31] Wang Y. Wang M. Ning F. Ren D. Tao J. Xie W. (2022). A novel role of BK potassium channel activity in preventing the development of kidney fibrosis. Kidney Int. 101, 945–962. 10.1016/j.kint.2021.11.033 34968553

[B32] Xia B. Shen X. He Y. Pan X. Liu F. Wang Y. (2021). SARS-CoV-2 envelope protein causes acute respiratory distress syndrome (ARDS)-like pathological damages and constitutes an antiviral target. Cell Res. 31, 847–860. 10.1038/s41422-021-00519-4 34112954 PMC8190750

[B33] Xu Z. Shi L. Wang Y. Zhang J. Huang L. Zhang C. (2020). Pathological findings of COVID-19 associated with acute respiratory distress syndrome. Lancet Respir. Med. 8, 420–422. 10.1016/S2213-2600(20)30076-X 32085846 PMC7164771

[B34] Xue J. Patergnani S. Giorgi C. Suarez J. Goto K. Bononi A. (2020). Asbestos induces mesothelial cell transformation via HMGB1-driven autophagy. Proc. Natl. Acad. Sci. U. S. A. 117, 25543–25552. 10.1073/pnas.2007622117 32999071 PMC7568322

[B35] Yang T. Xu C. (2017). Physiology and pathophysiology of the intrarenal renin-angiotensin system: An update. J. Am. Soc. Nephrol. 28, 1040–1049. 10.1681/ASN.2016070734 28255001 PMC5373463

[B36] Zhang P. Zhu L. Cai J. Lei F. Qin J. Xie J. (2020). Association of inpatient use of angiotensin-converting enzyme inhibitors and angiotensin II receptor blockers with mortality among patients with hypertension hospitalized with COVID-19. Circ. Res. 126, 1671–1681. 10.1161/CIRCRESAHA.120.317134 32302265 PMC7265882

[B37] Zhang X. Yu J. Pan L. Jiang H. (2020). ACEI/ARB use and risk of infection or severity or mortality of COVID-19: A systematic review and meta-analysis. Pharmacol. Res. 158, 104927. 10.1016/j.phrs.2020.104927 32422341 PMC7227582

[B38] Zhao Z. Hu Z. Zeng R. Yao Y. (2020). HMGB1 in kidney diseases. Life Sci. 259, 118203. 10.1016/j.lfs.2020.118203 32781069

[B39] Zheng M. Karki R. Williams E. P. Yang D. Fitzpatrick E. Vogel P. (2021). TLR2 senses the SARS-CoV-2 envelope protein to produce inflammatory cytokines. Immunol 22, 829–838. 10.1038/s41590-021-00937-x PMC8882317 33963333

[B40] Zou H. Ming B. Li J. Xiao Y. Lai L. Gao M. (2021). Extracellular HMGB1 contributes to the chronic cardiac allograft vasculopathy/fibrosis by modulating TGF-β1 signaling. Front. Immunol. 12, 641973. 10.3389/fimmu.2021.641973 33777037 PMC7988222

