# Supplementary material for: Olmesartan alleviates SARS-CoV-2 envelope protein induced renal fibrosis by regulating HMGB1 release and autophagic degradation of TGF-β1
Source: Front Pharmacol. 2023 May 15;14:1187818. doi: 10.3389/fphar.2023.1187818 (PMC10225711; doi:10.3389/fphar.2023.1187818)

Supplementary Material - original images

Figure 1. Multiple organ damage induced by E protein.

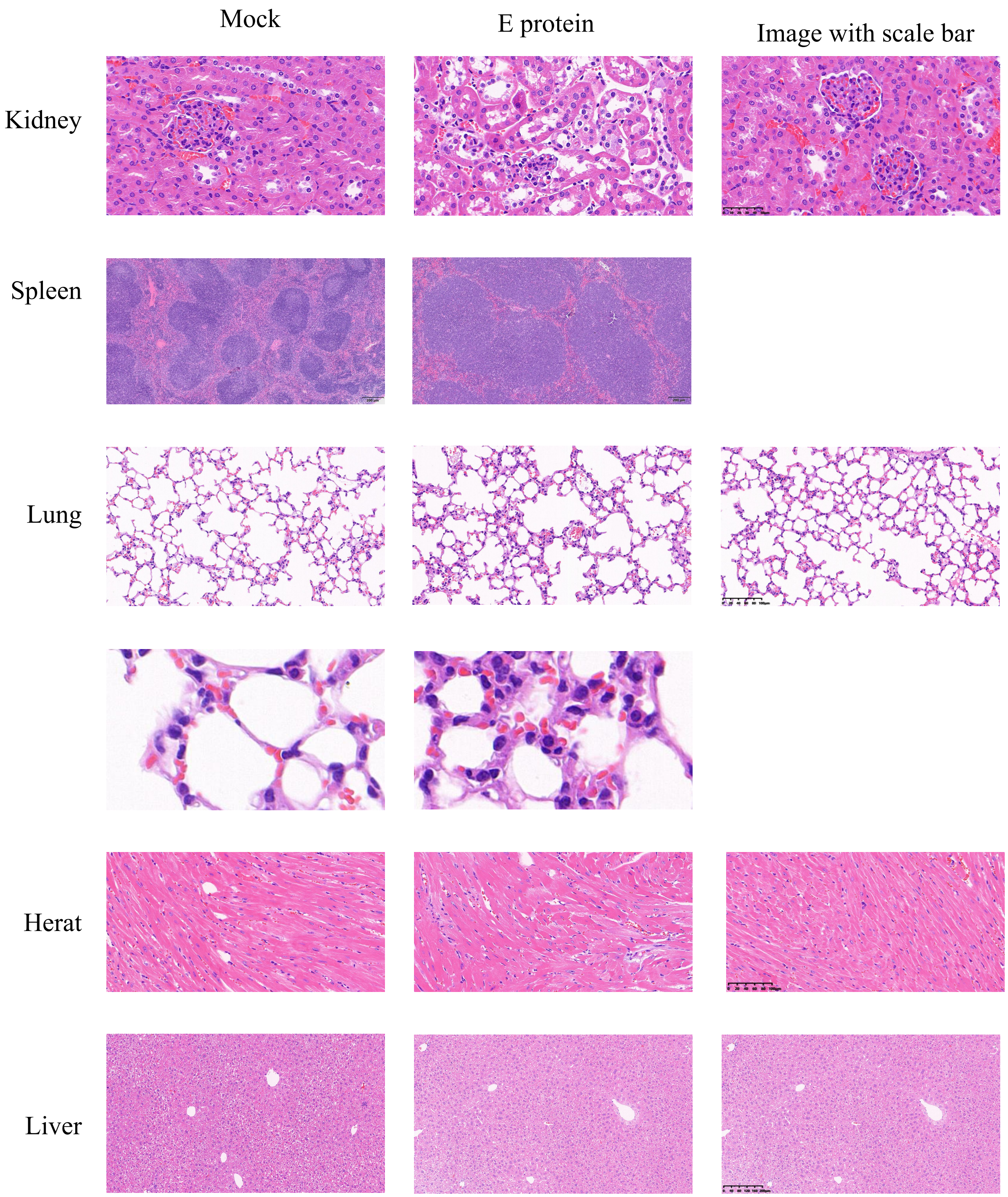

Figure 2. Olmesartan alleviates renal damage caused by E protein.

F

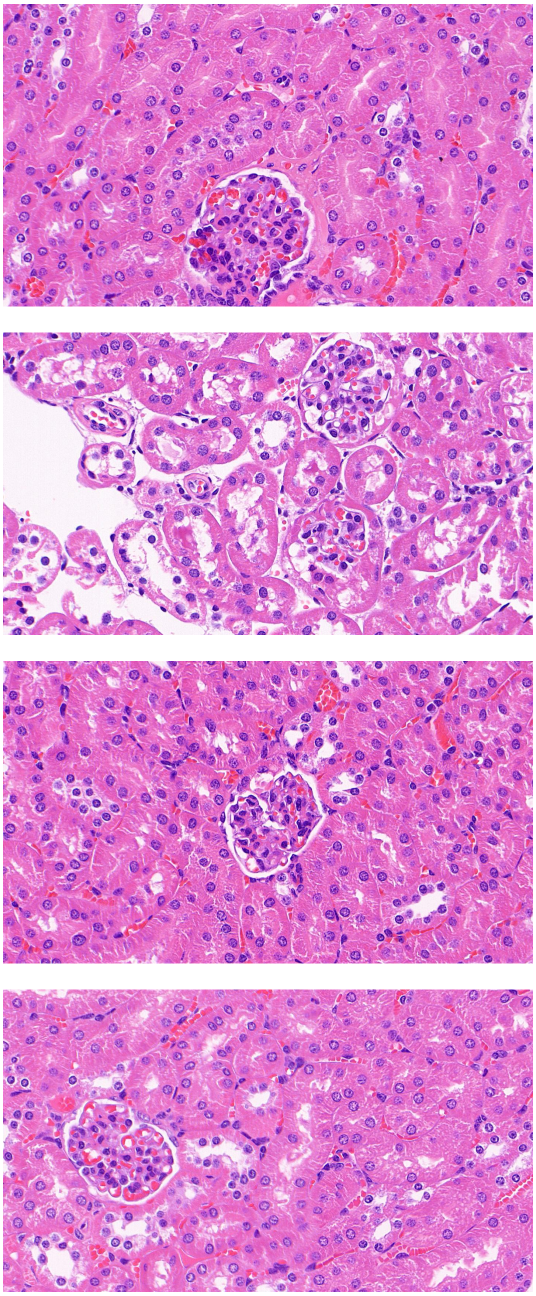

Figure 3. Olmesartan improves E protein-induced renal fibrosis.

D

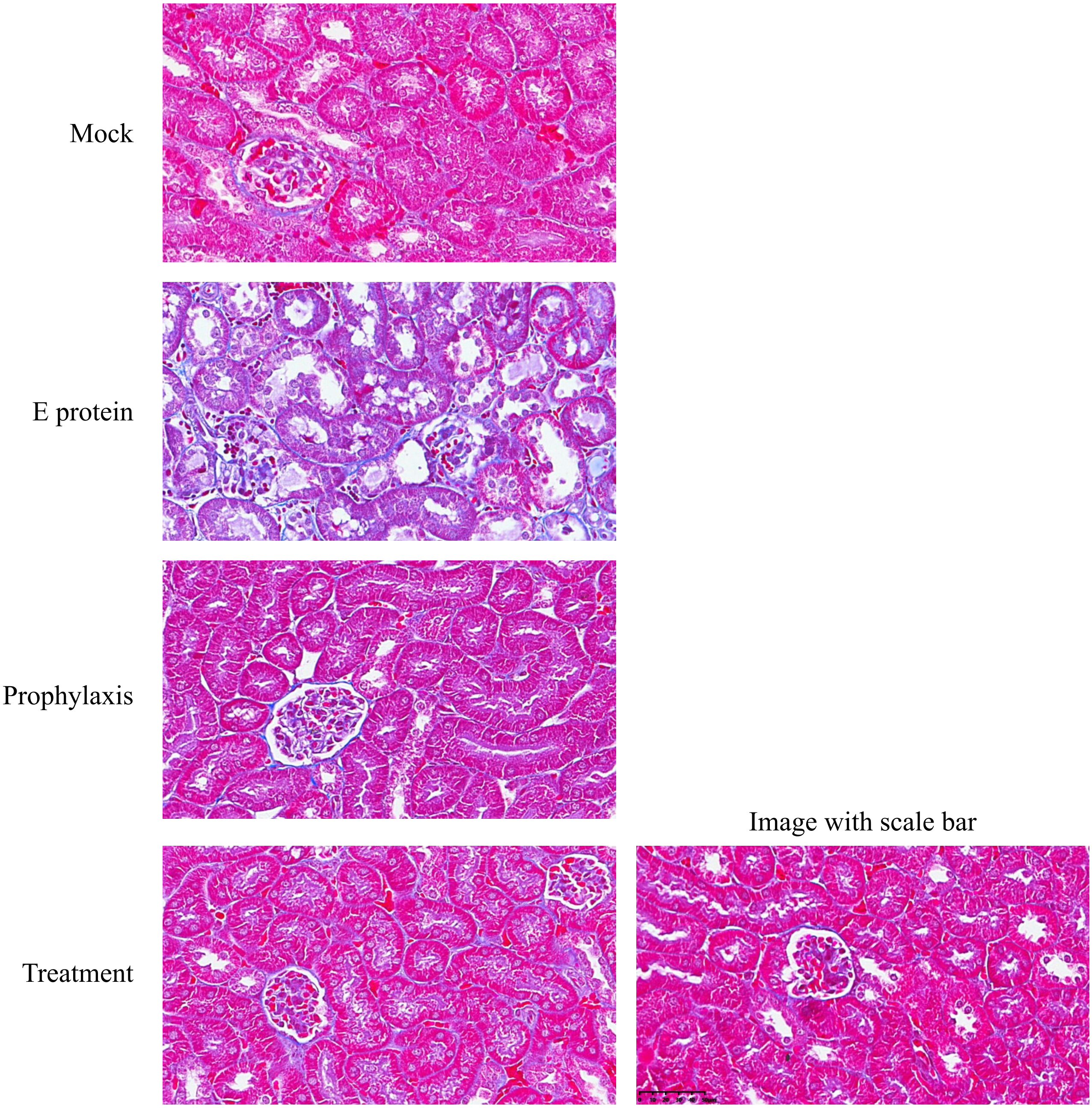

Original images

Supplementary Figure 3. Effect of prophylactic administration of olmesartan on E protein induced damage.

Kidney

Mock

E protein

Prophylaxis

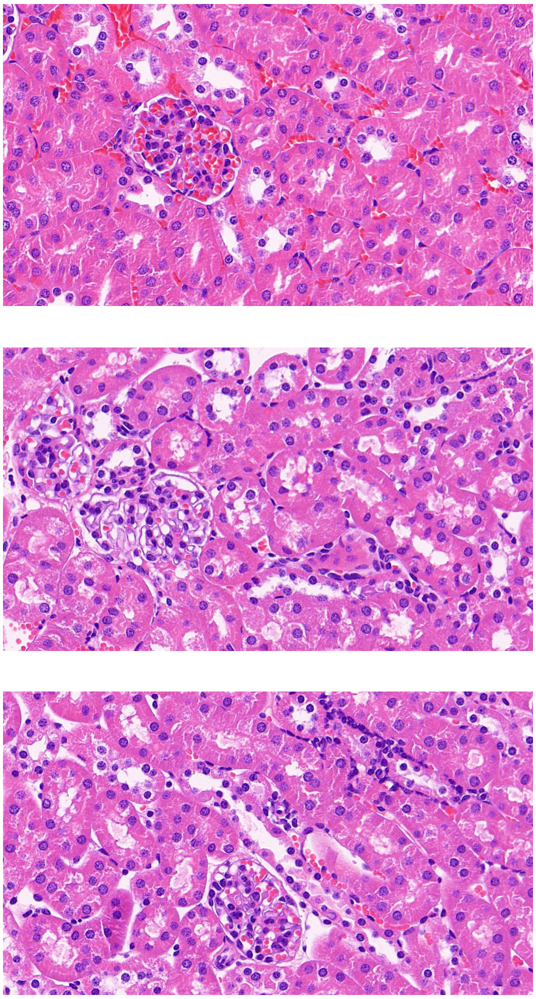

Original images

Supplementary Figure 4. Comparison of histopathological features.

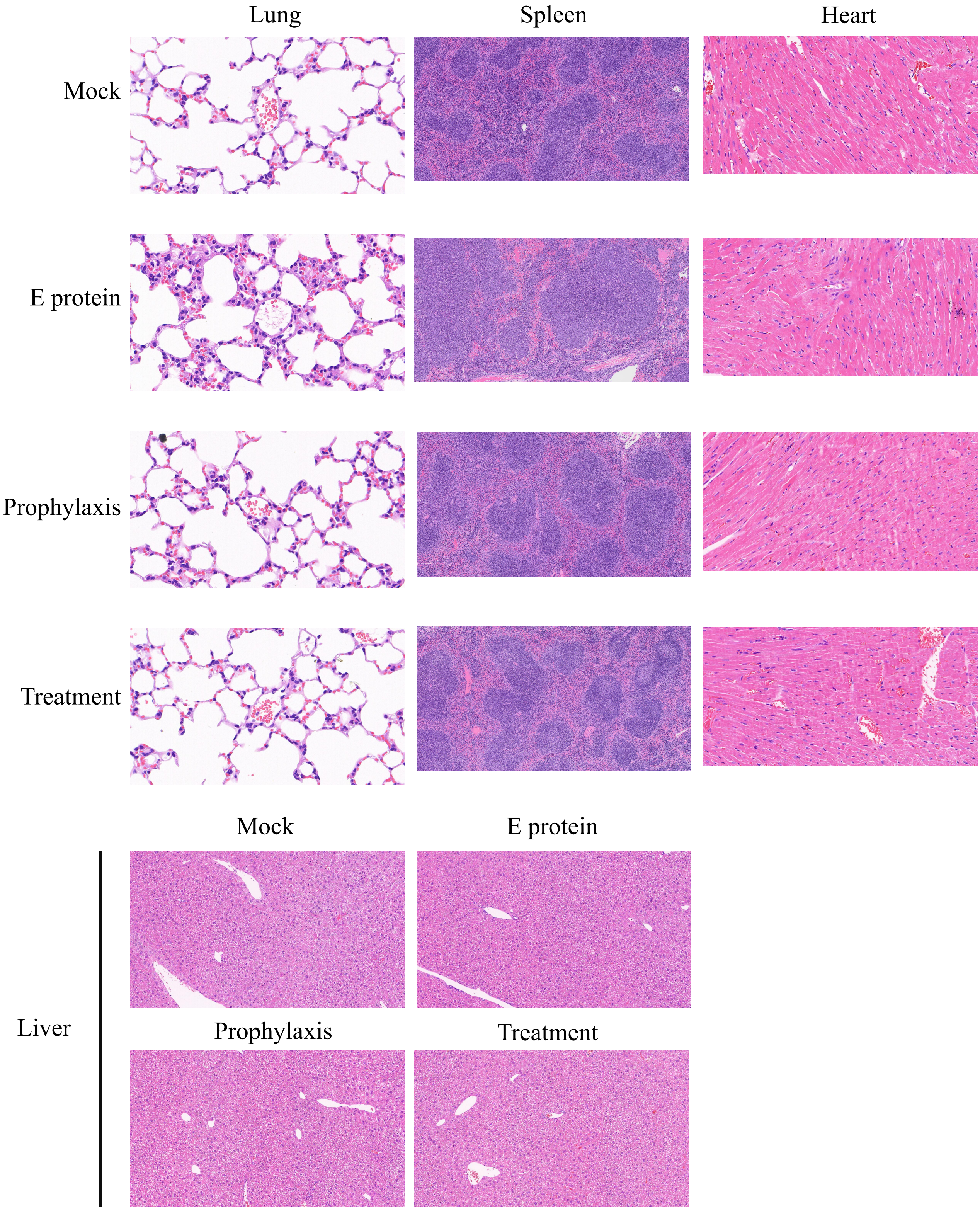

Figure 5. Olmesartan up-regulates the cytoplasmic level of HMGB1.

D

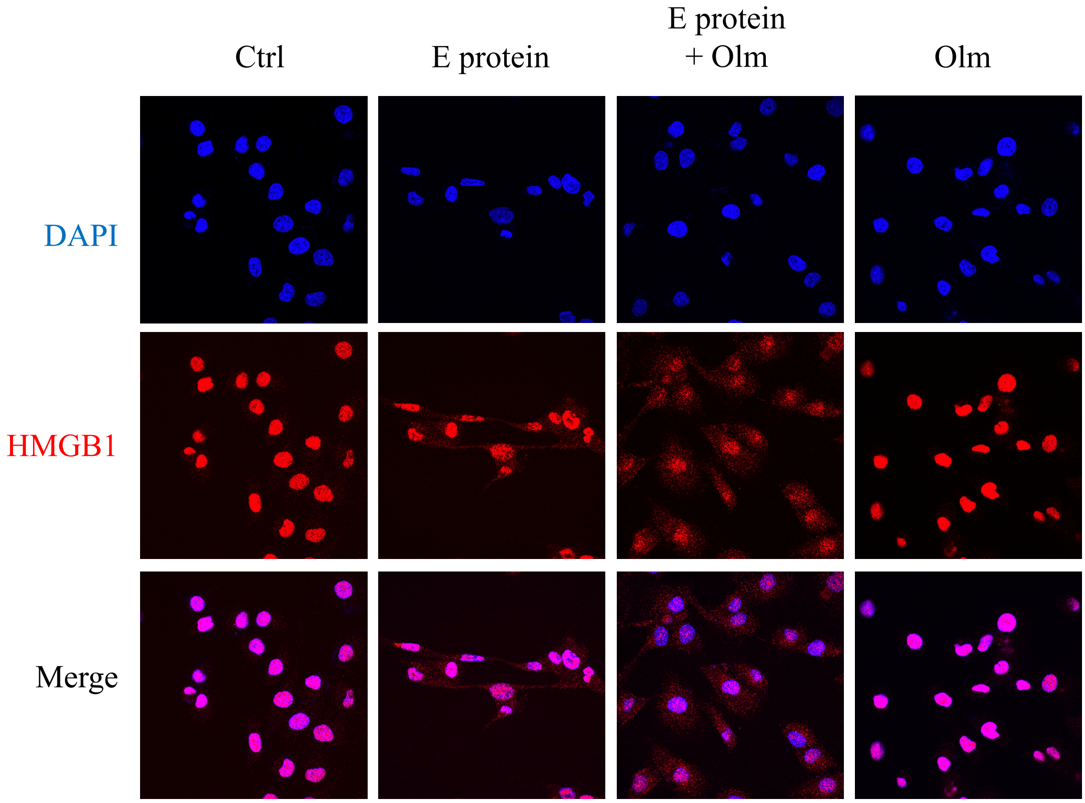

Image with scale bar

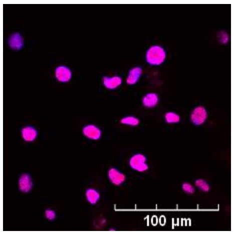

Figure 6. Olmesartan promotes autophagic degradation of TGF-β1 in renal tubular epithelial cells.

D

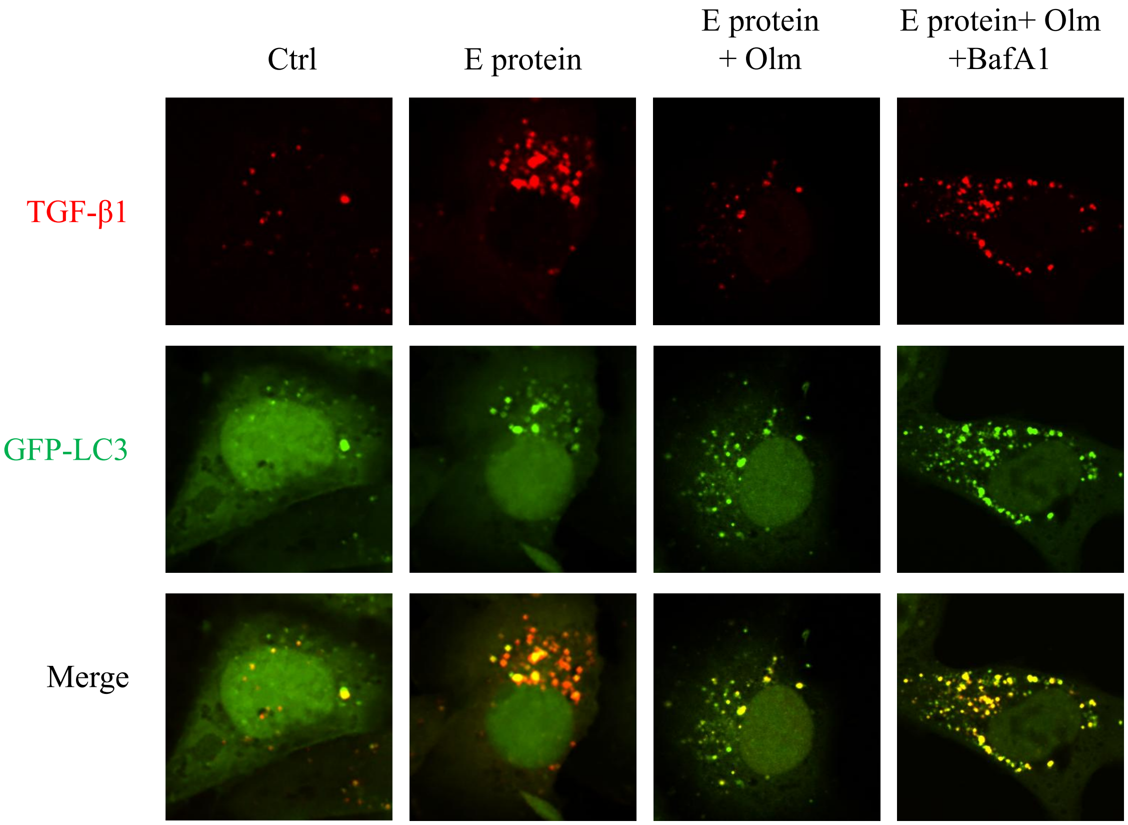

Image with scale bar

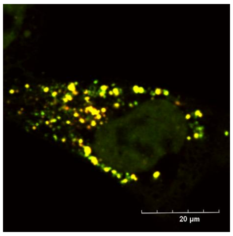

In the western blotting images, different gels were used because the molecular weight of target proteins were too close to be separated. In addition, the loading samples of different gels were the same, and electrophoresis was performed at the same time.

The image displayed in the article has been marked with a dashed box.

Figure 3. Olmesartan improves E protein-induced renal fibrosis.

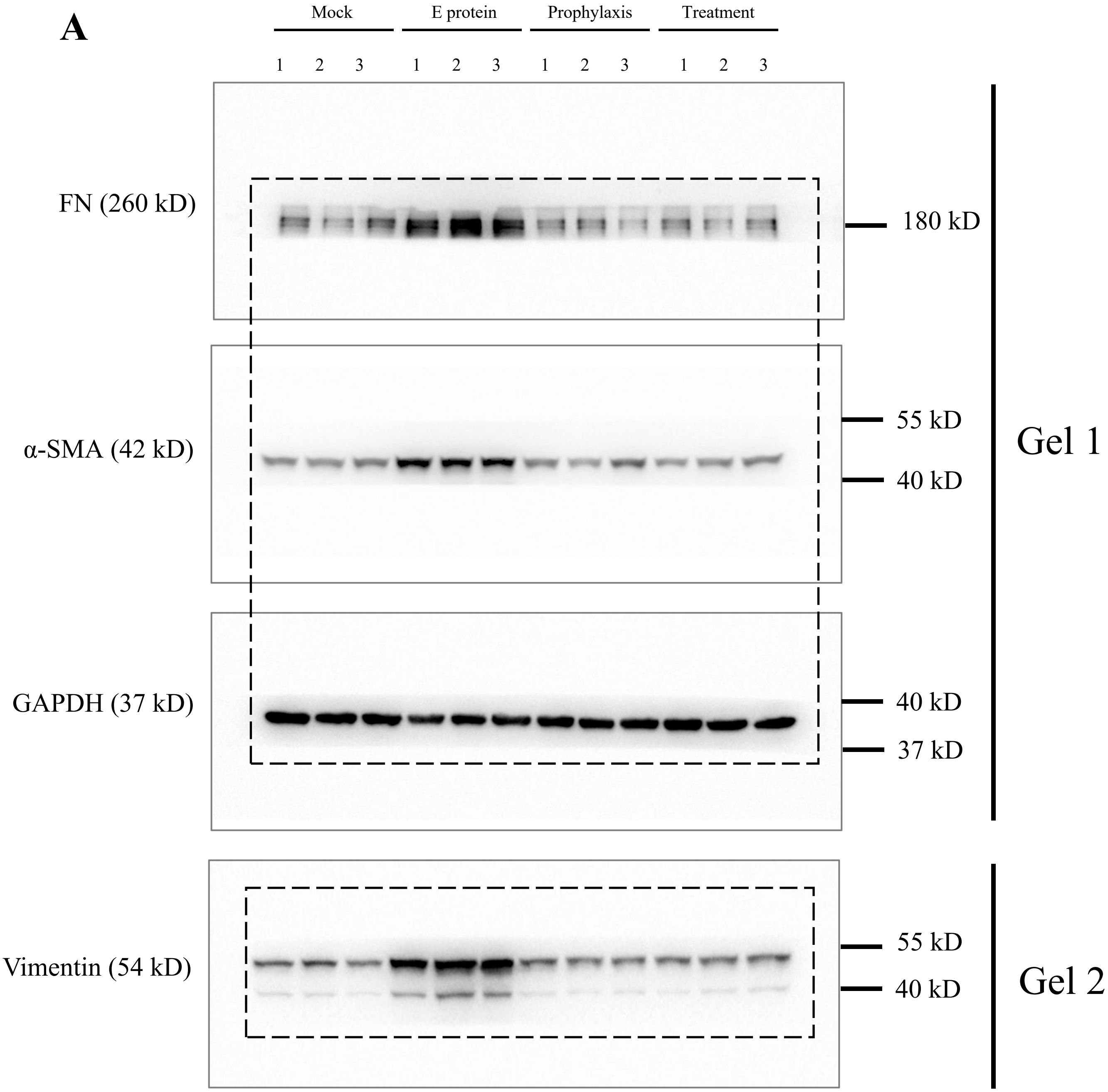

Figure 3. Olmesartan improves E protein-induced renal fibrosis.

E

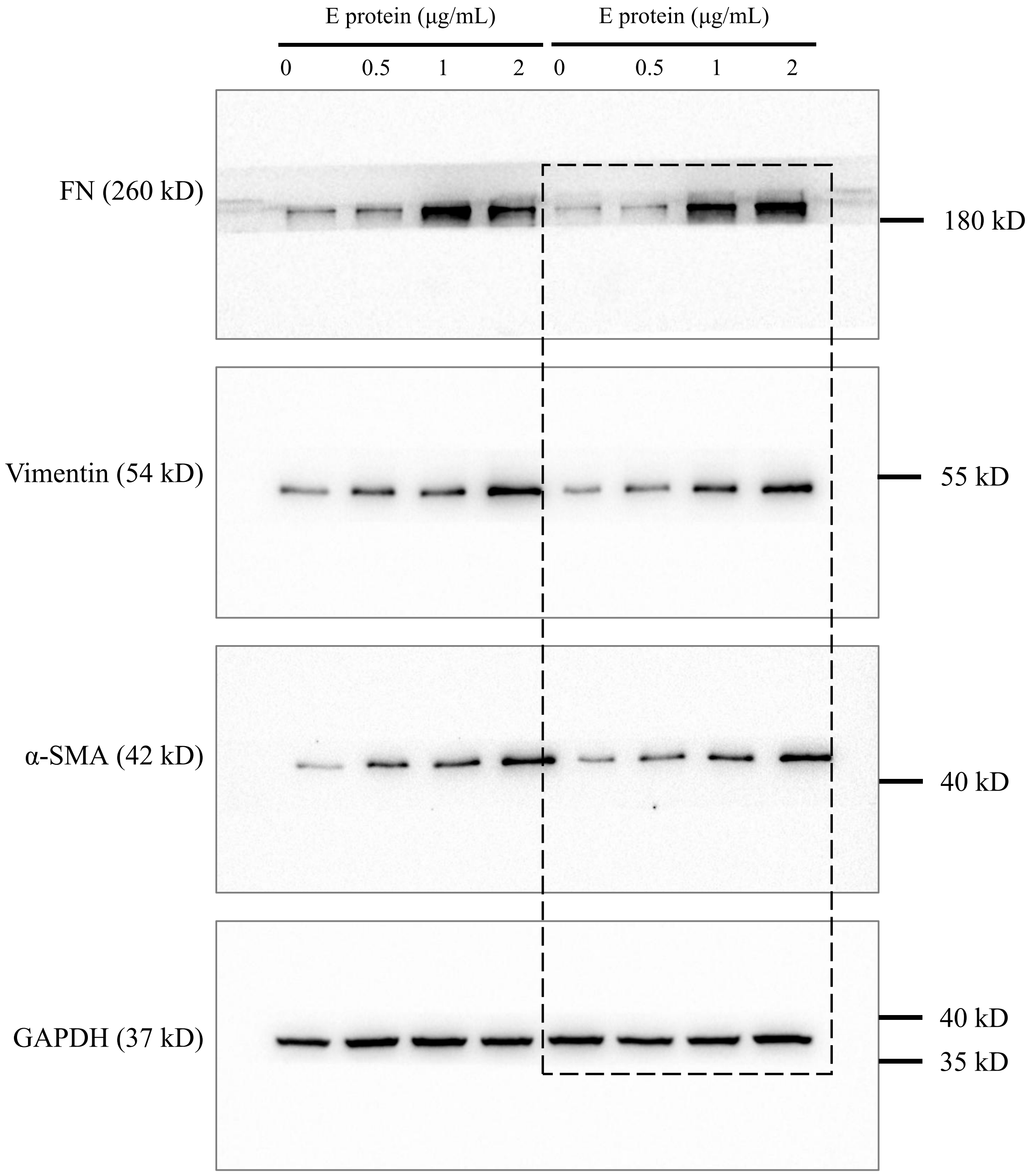

Figure 3. Olmesartan improves E protein-induced renal fibrosis.

G

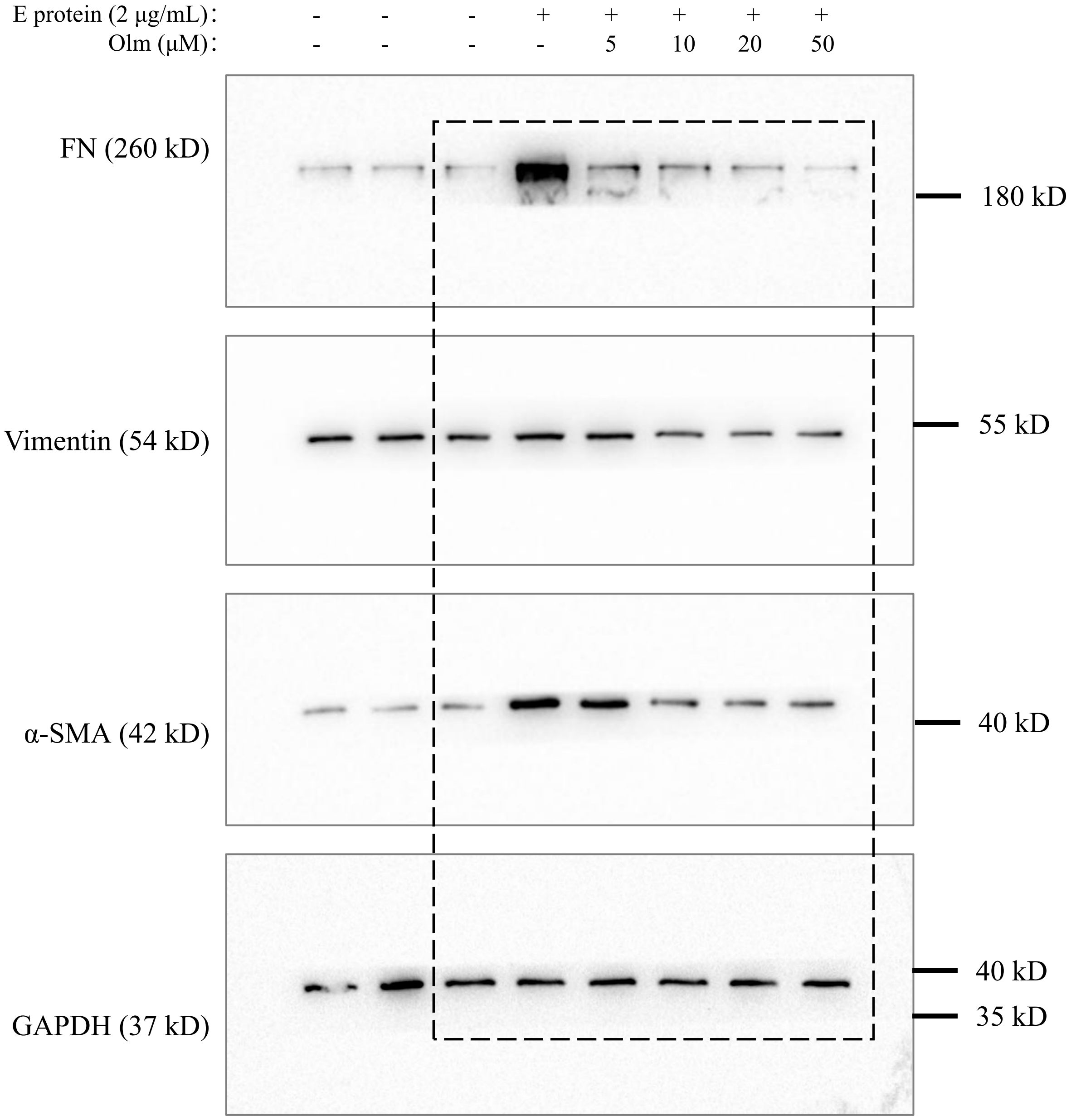

Figure 5. Olmesartan up-regulates the level of cytoplasmic HMGB1.

A

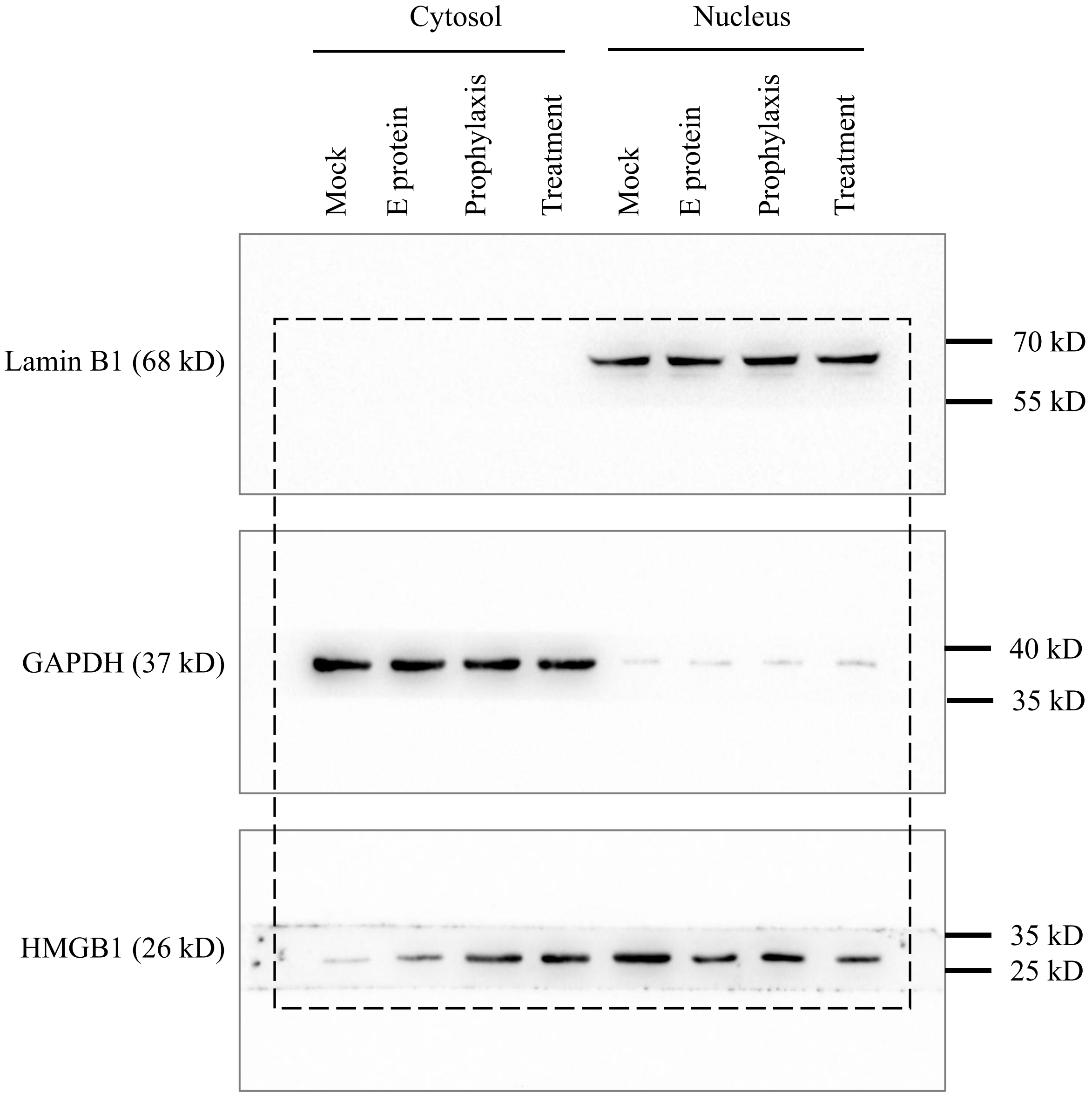

Figure 5. Olmesartan up-regulates the level of cytoplasmic HMGB1.

C

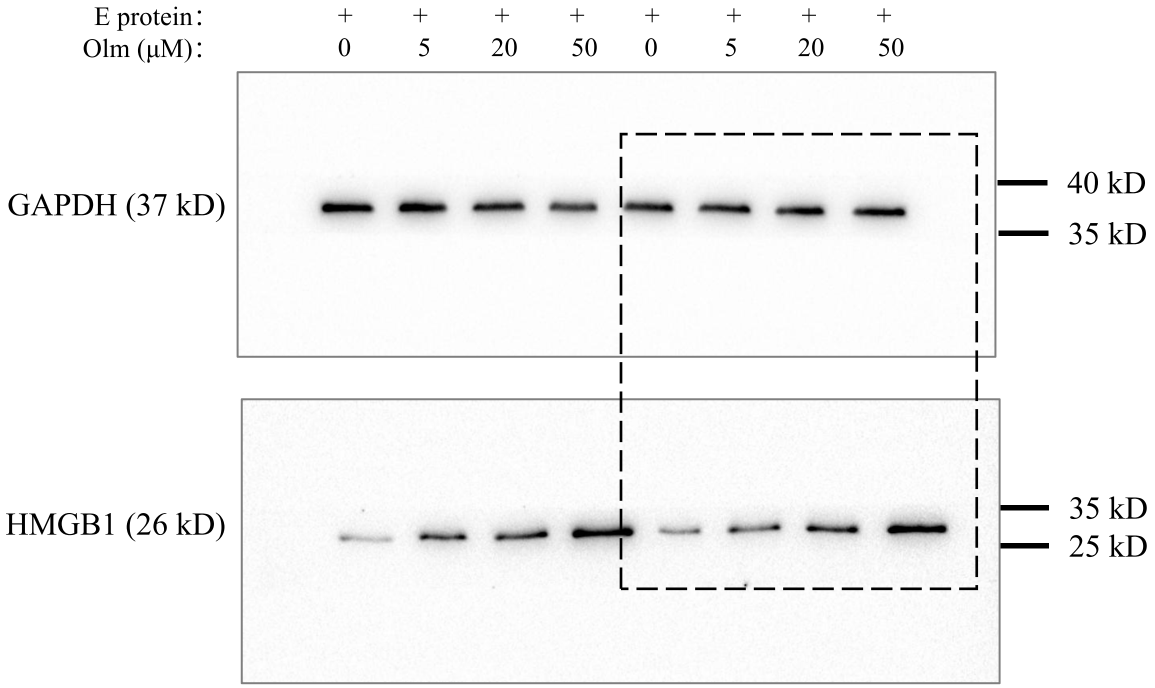

Figure 6. Olmesartan promotes autophagic degradation of TGF-β1 in renal tubular epithelial cells.

A

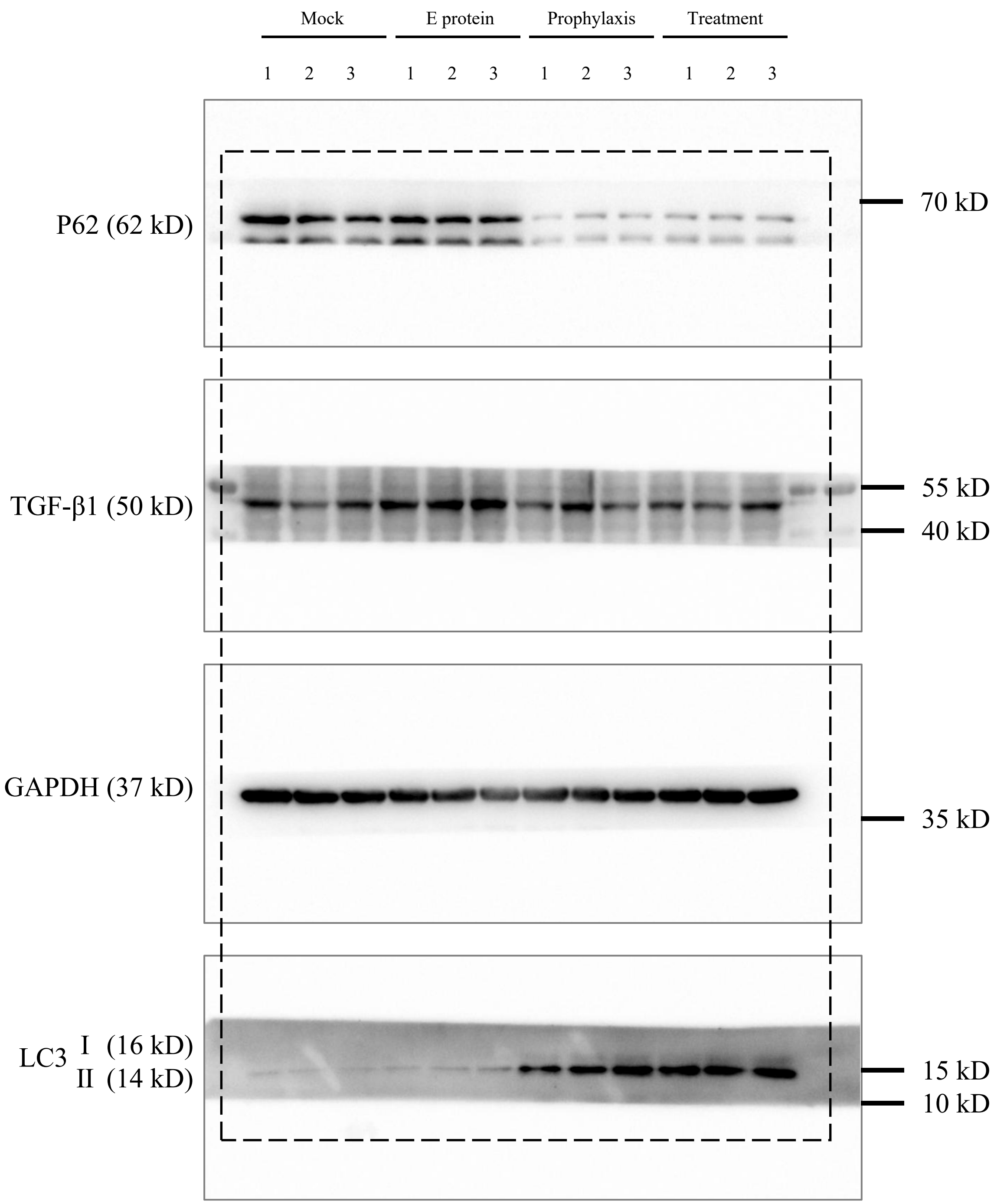

Figure 6. Olmesartan promotes autophagic degradation of TGF-β1 in renal tubular epithelial cells.

C

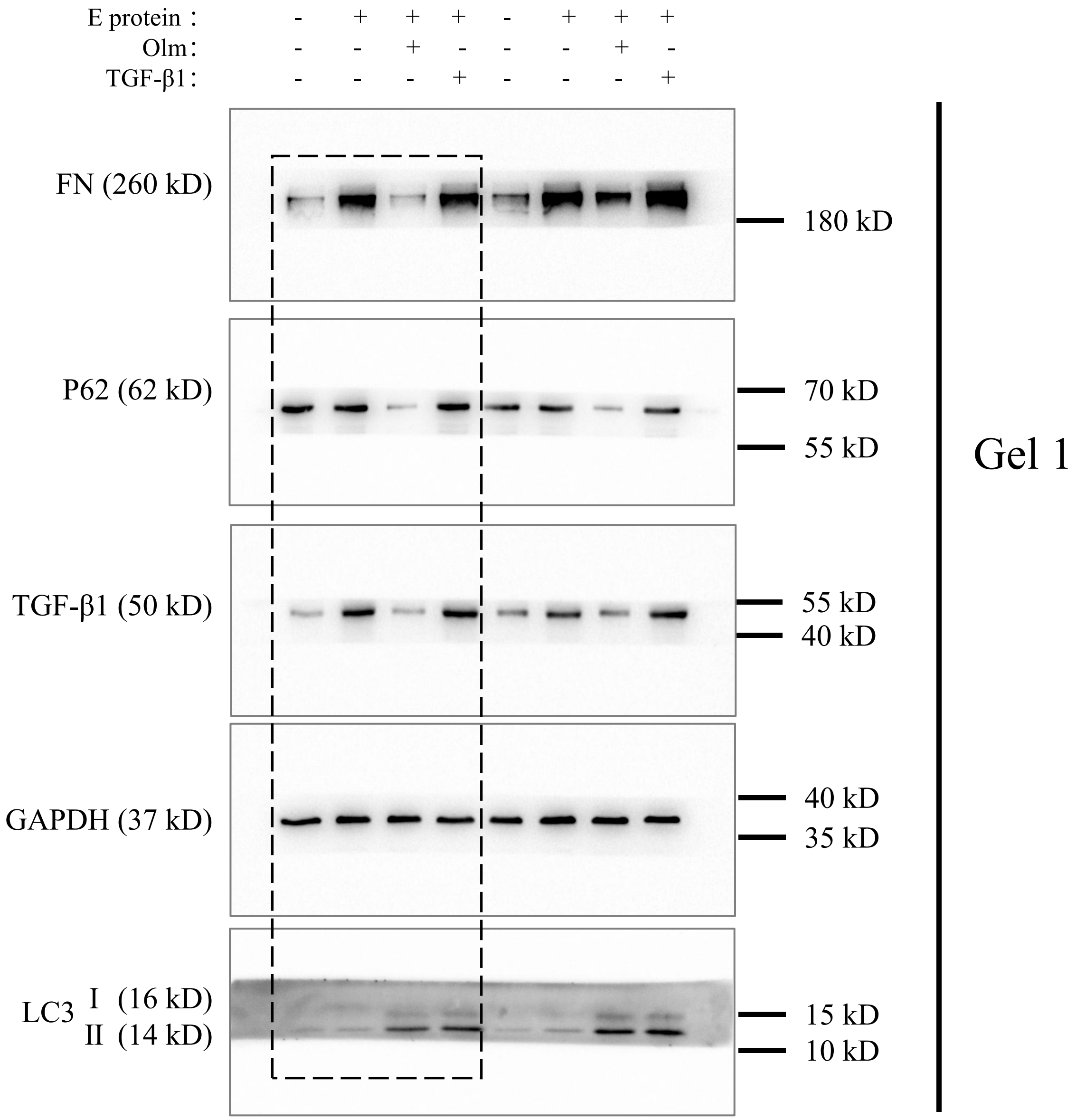

Figure 6. Olmesartan promotes autophagic degradation of TGF-β1 in renal tubular epithelial cells.

C

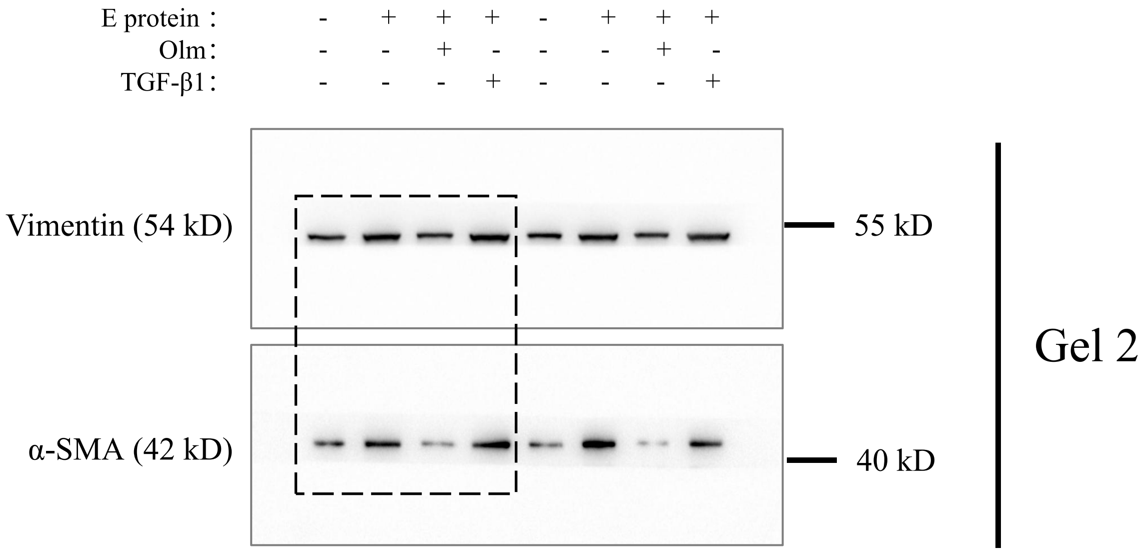

Figure 7. The regulation of Olmesartan on autophagy is mediated by HMGB1.

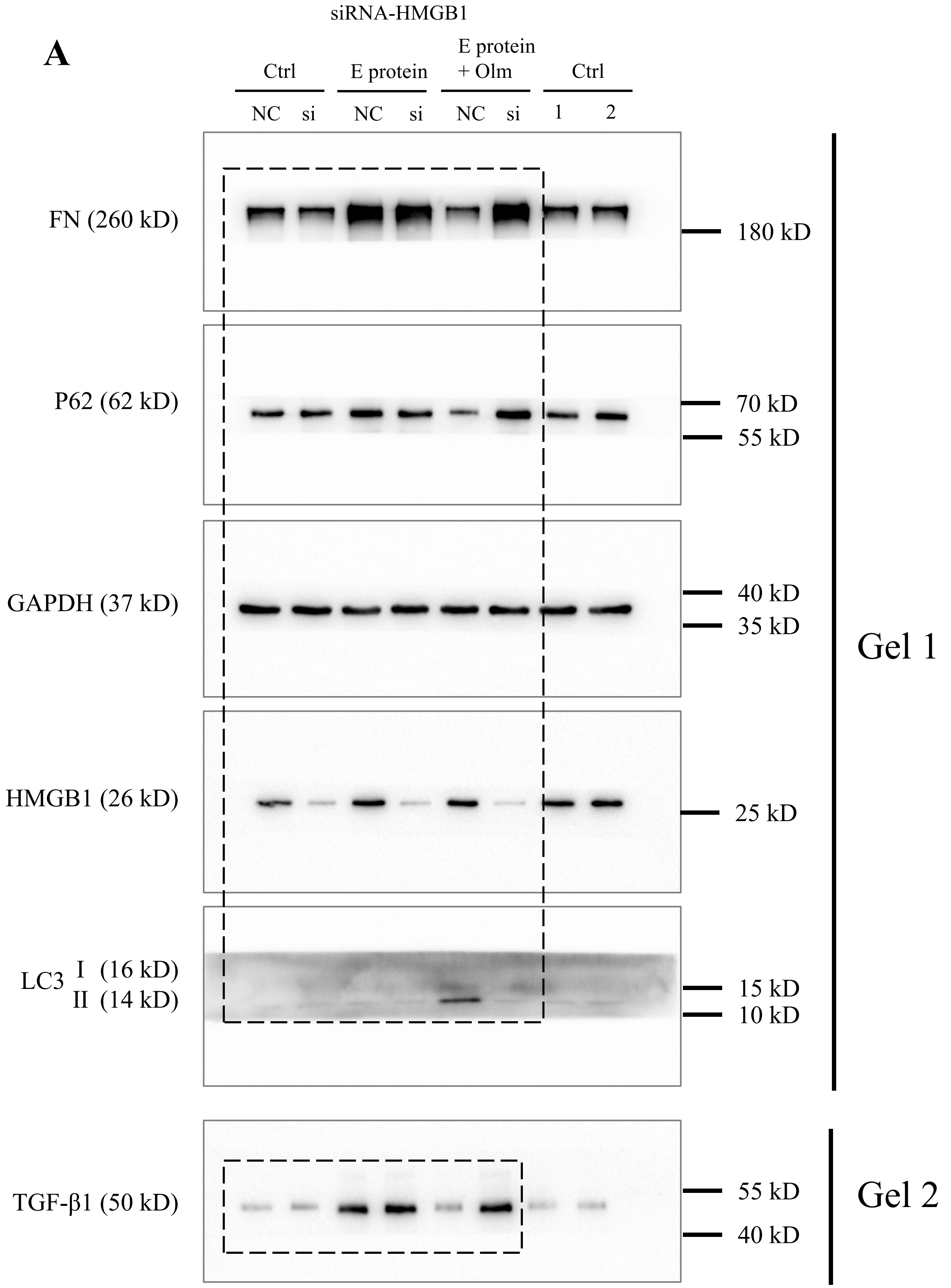

Figure 7. The regulation of Olmesartan on autophagy is mediated by HMGB1.

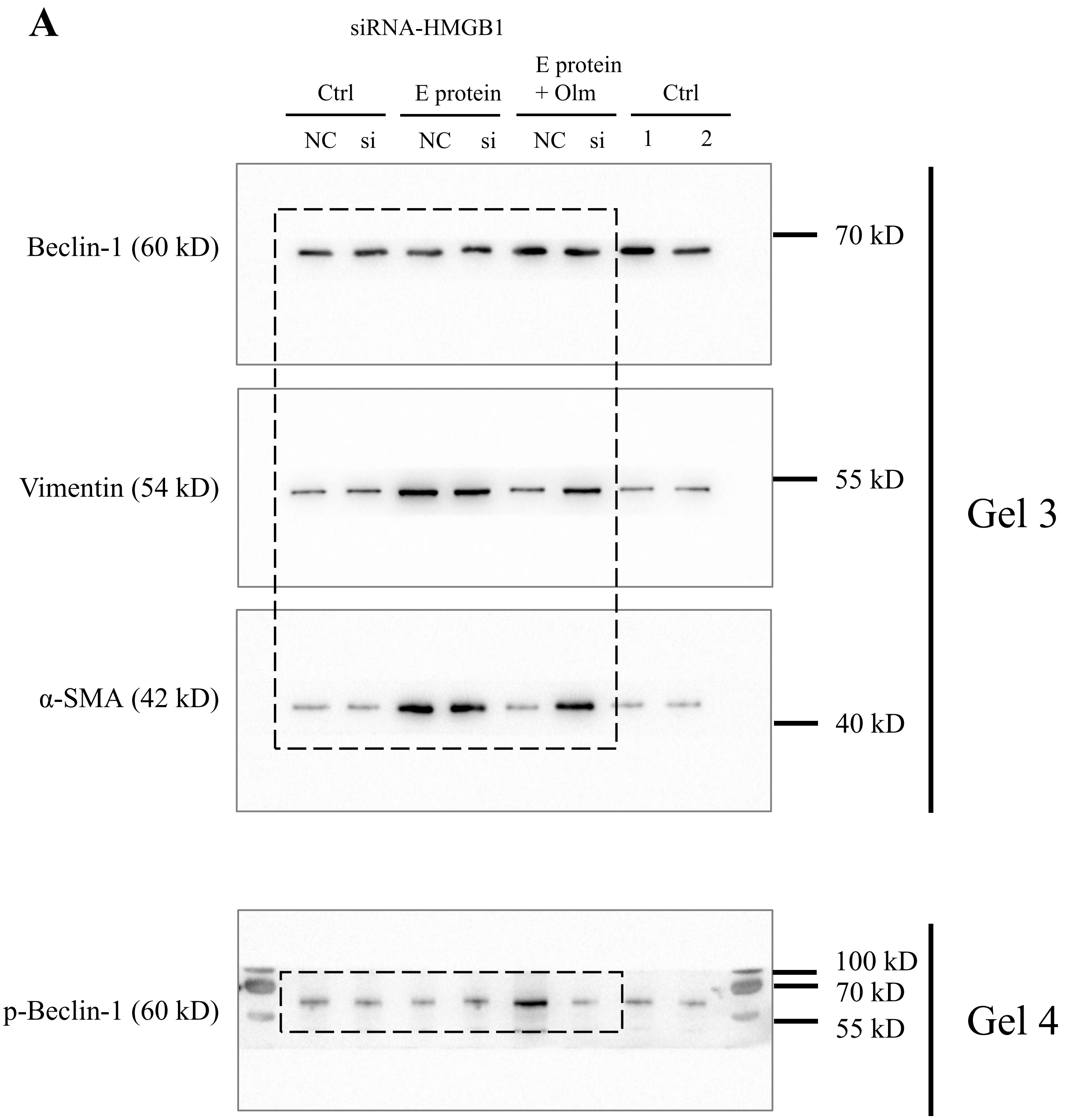

Ctrl 1,2 without any treatments.

Supplementary Figure 1. Drug screening in vitro.

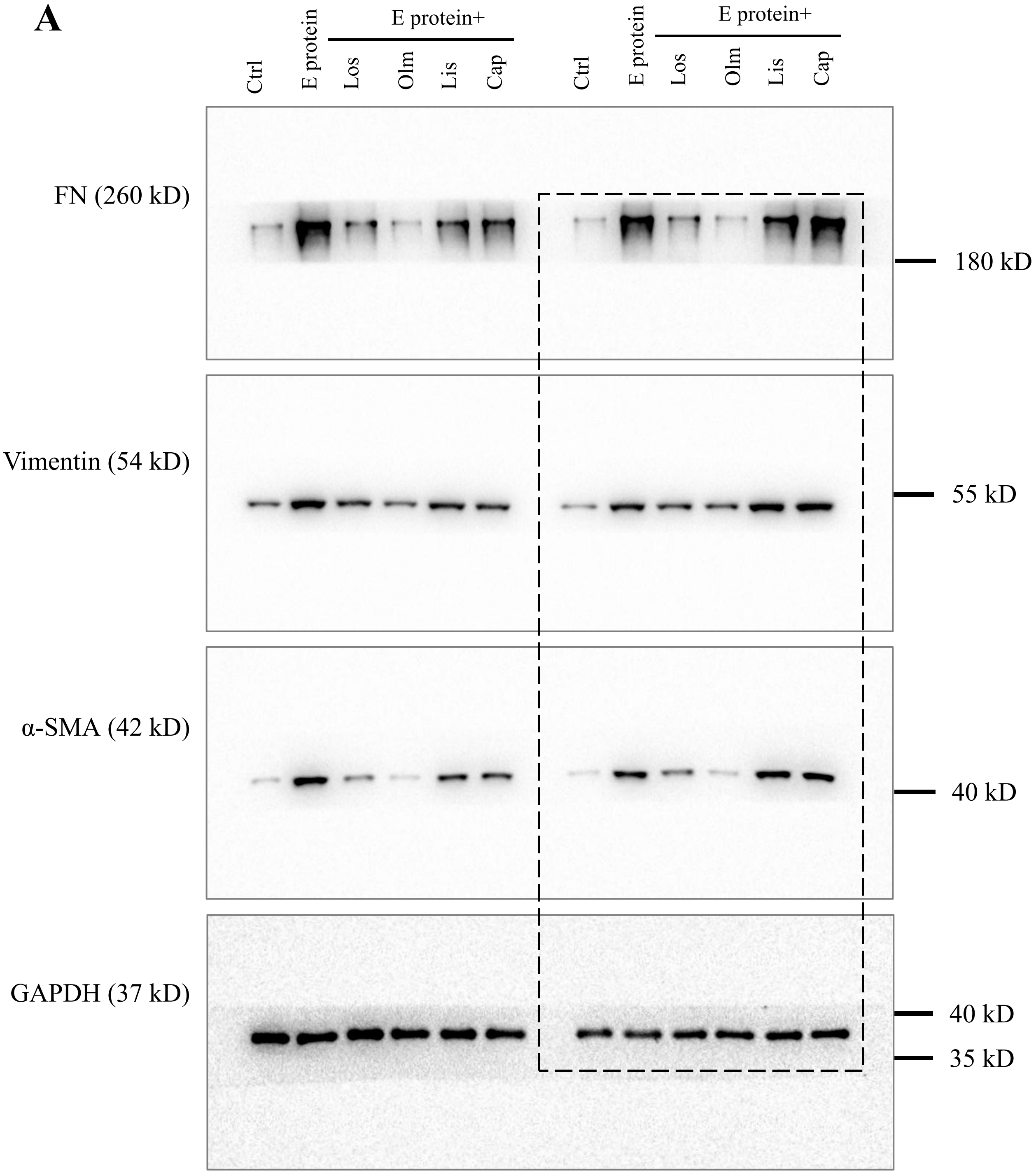

Los: losartan; Olm: olmesartan;
Lis: lisinopril; Cap: captopril.

Supplementary Figure 2. Purification and validation of SARS-CoV-2 E proteins.

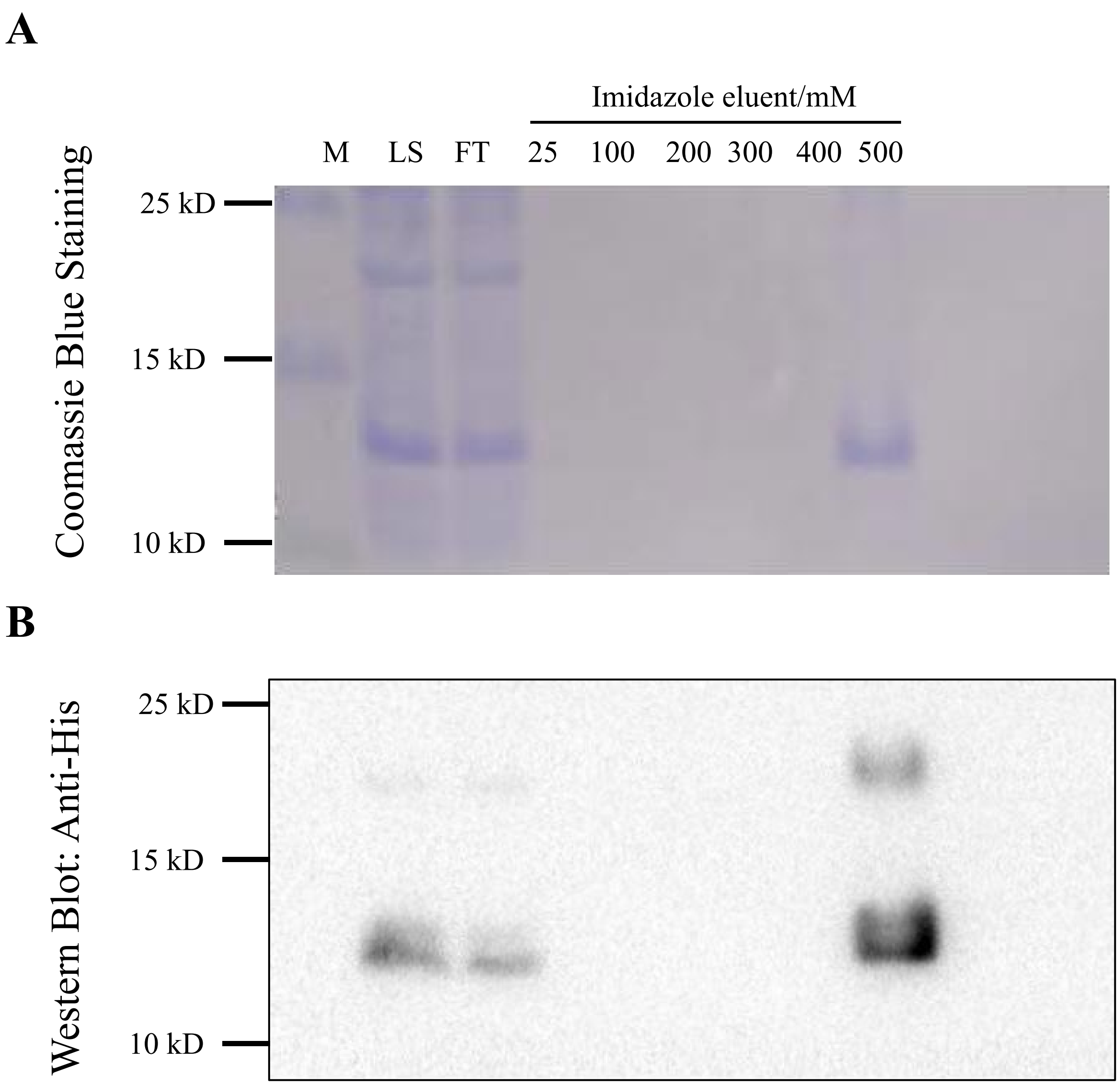

M: maker; LS: lysate; FT: flowthrough fluid.

Supplementary Figure 5. Olmesartan inhibits the activation of TGF-β1/Smad2/3 pathway.

A

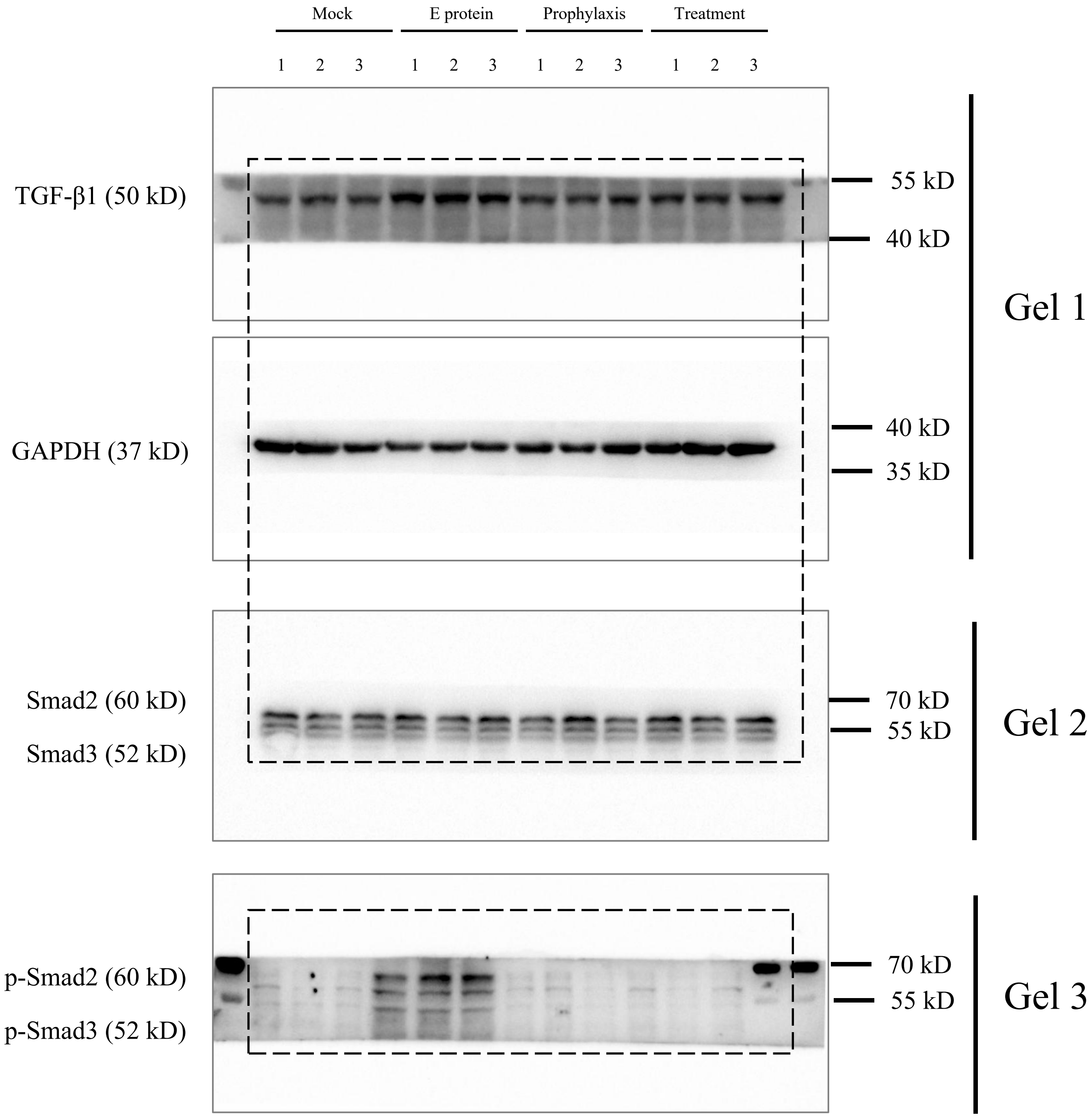

Original images

Supplementary Figure 5. Olmesartan inhibits the activation of TGF-β1/Smad2/3 pathway.

C

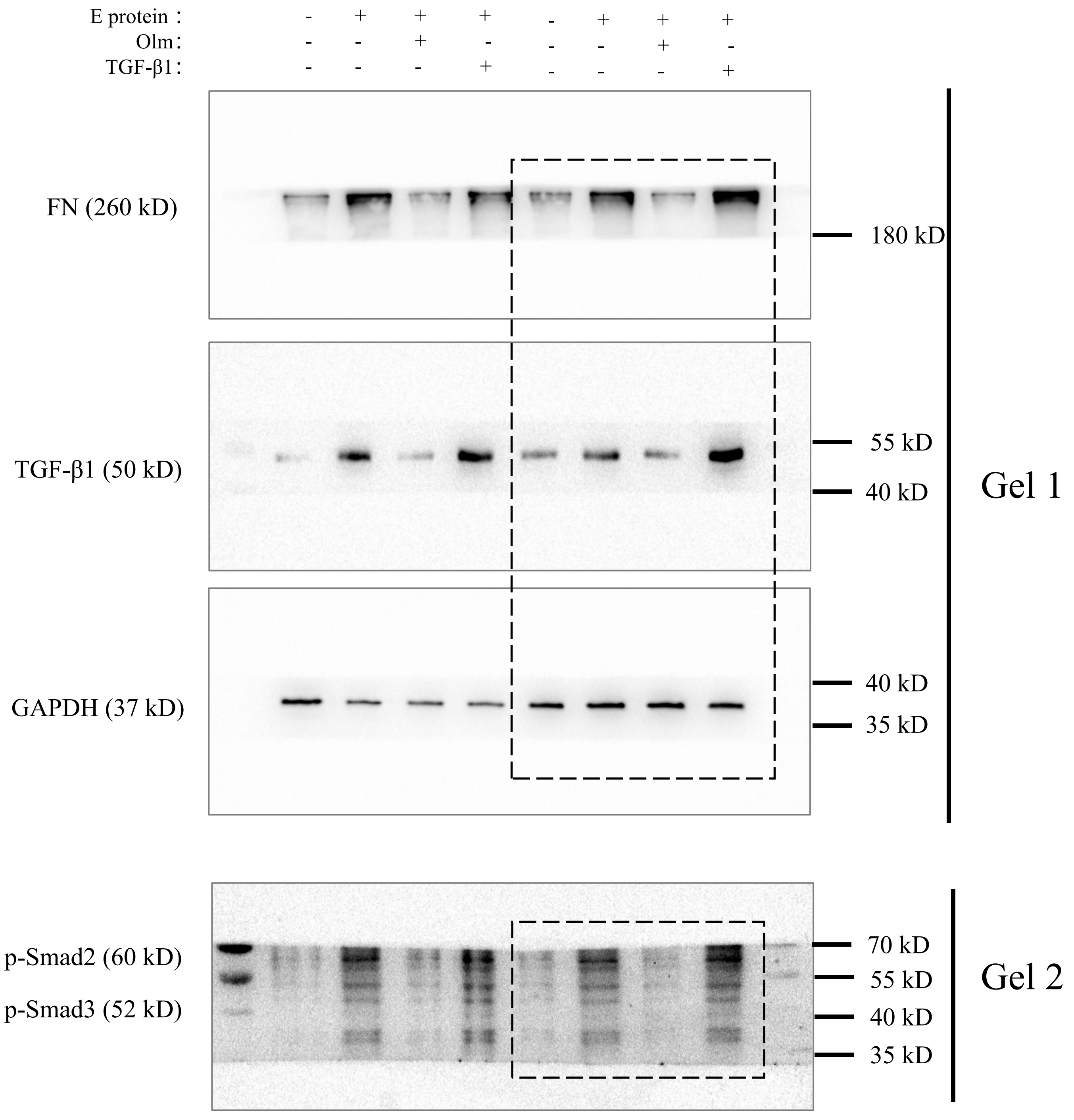

Supplementary Figure 5. Olmesartan inhibits the activation of TGF-β1/Smad2/3 pathway.

C

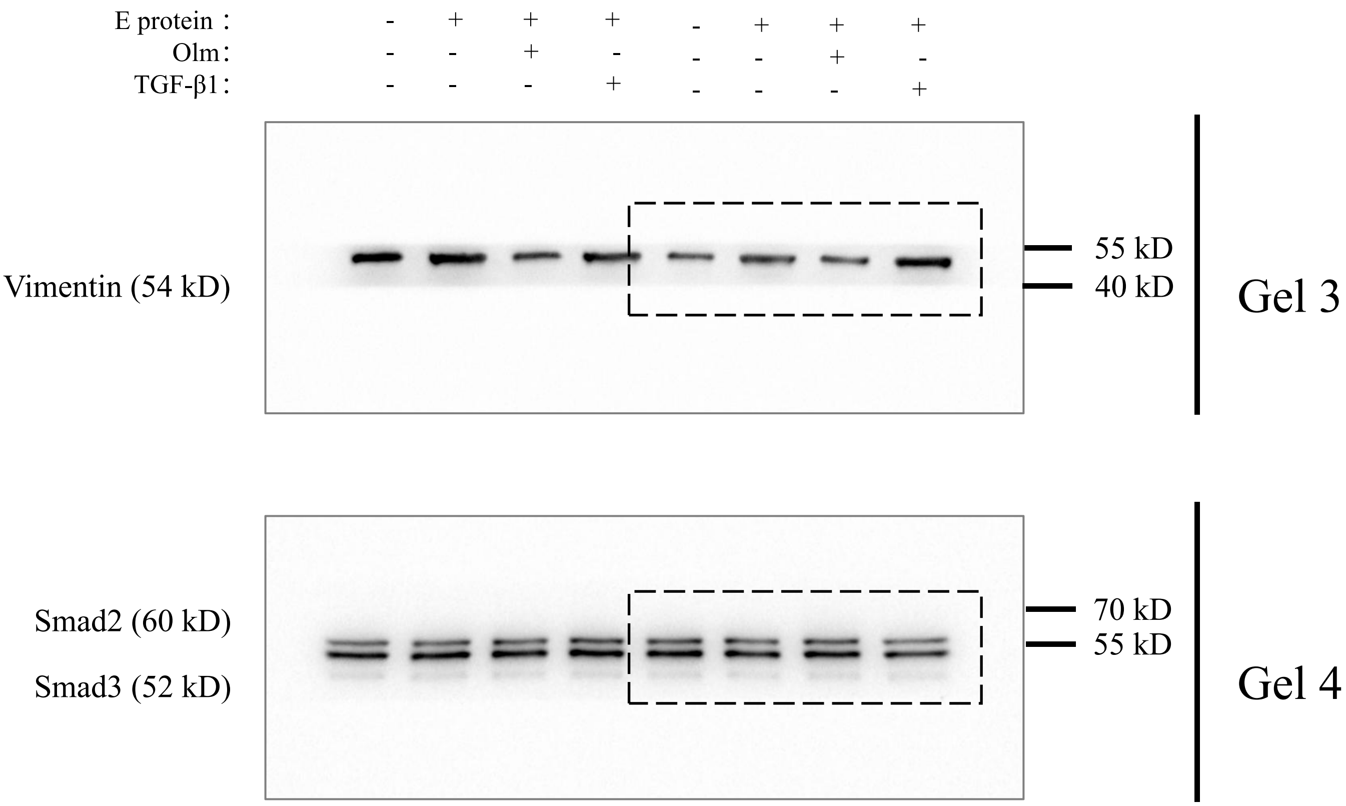

Supplementary Figure 6. Regulation of HMGB1 by Olmesartan in HK-2.

A

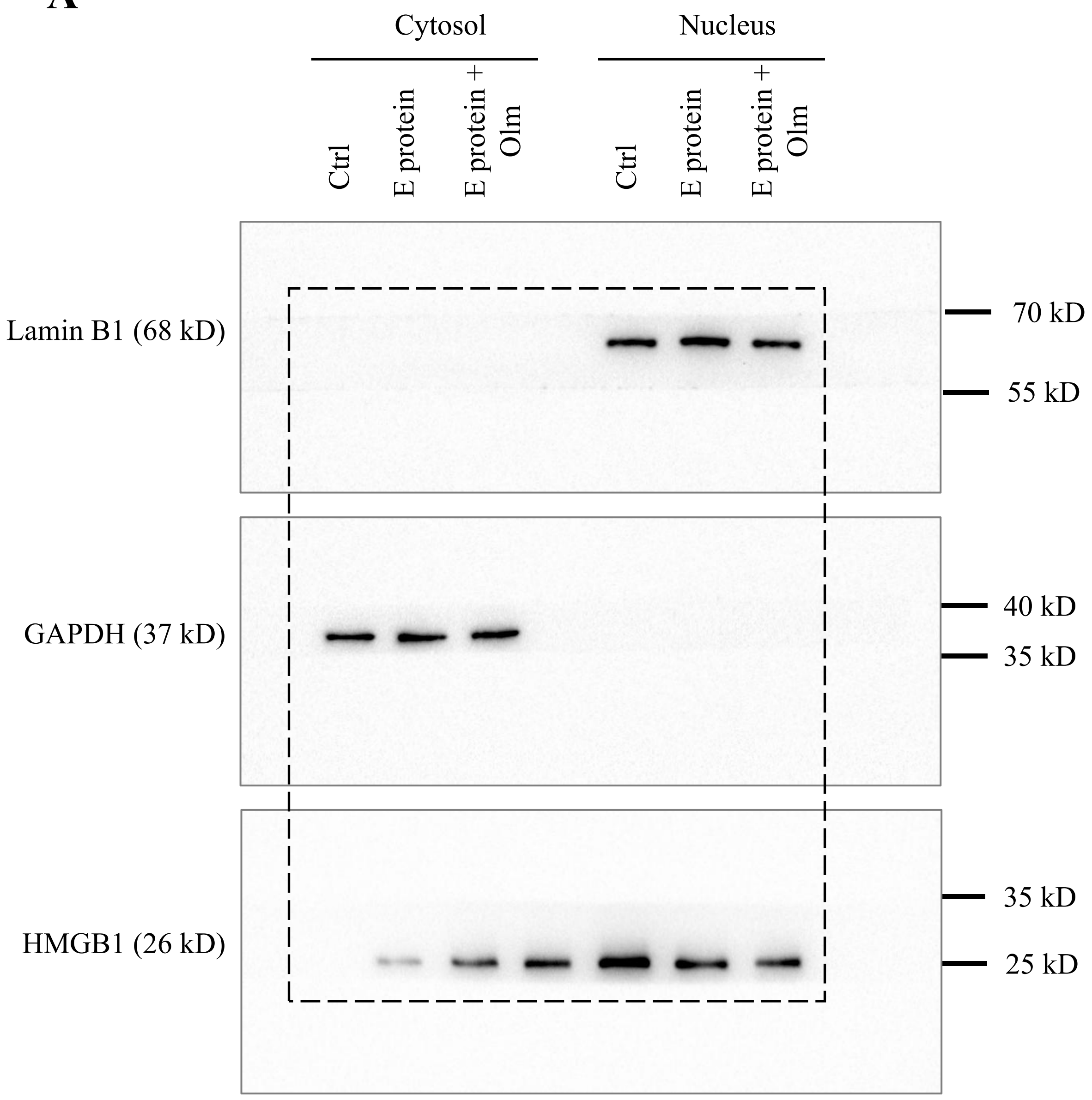

Supplementary Figure 7. TGF-β1 is degraded by autophagy.

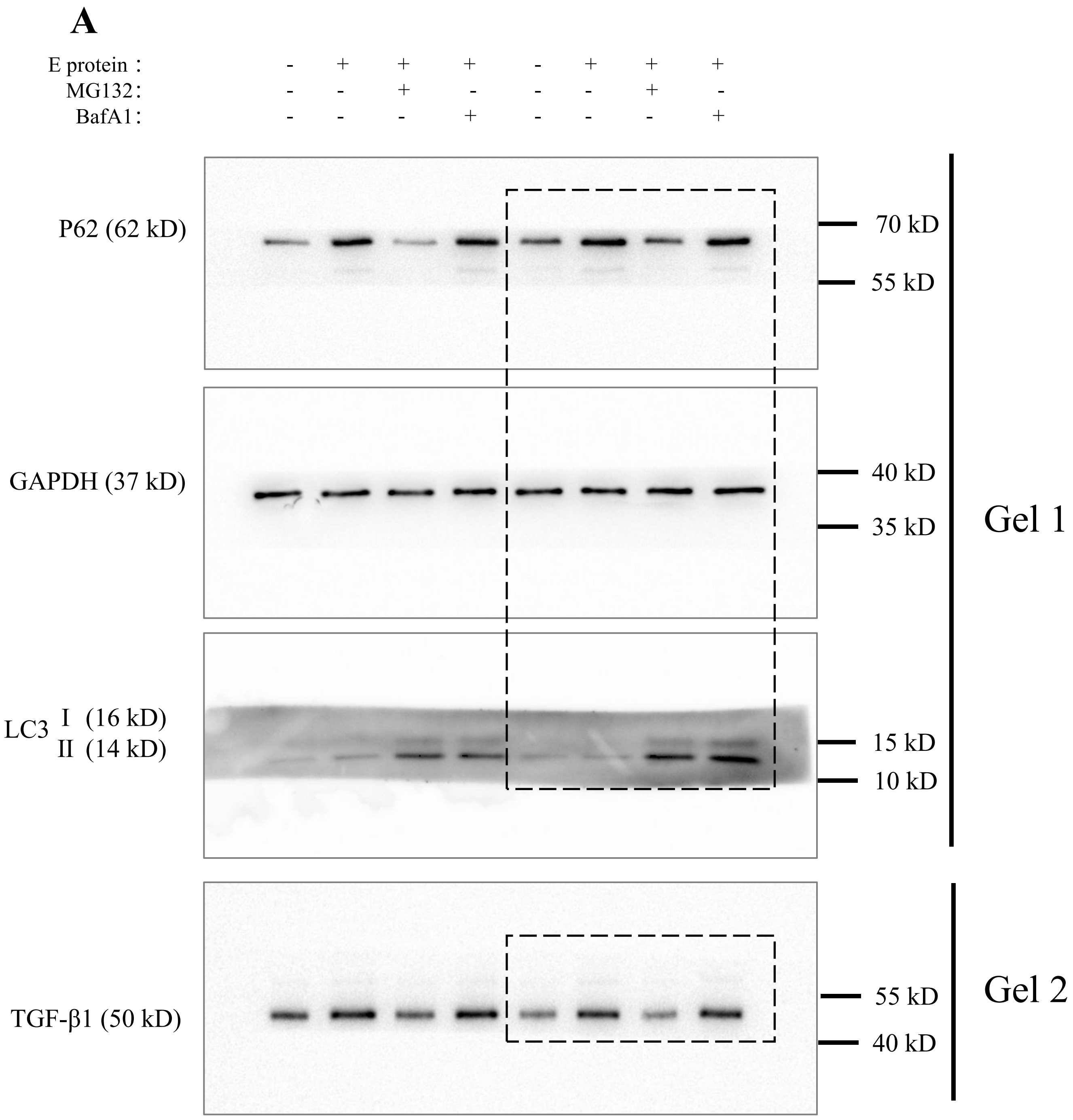

Supplement: Supplementary file 1 [file DataSheet2.pdf]
