# Supplementary material for: Olmesartan alleviates SARS-CoV-2 envelope protein induced renal fibrosis by regulating HMGB1 release and autophagic degradation of TGF-β1
Source: Front Pharmacol. 2023 May 15;14:1187818. doi: 10.3389/fphar.2023.1187818 (PMC10225711; doi:10.3389/fphar.2023.1187818)
Supplement: Supplementary file 3 [file DataSheet1.pdf]

Supplementary Material

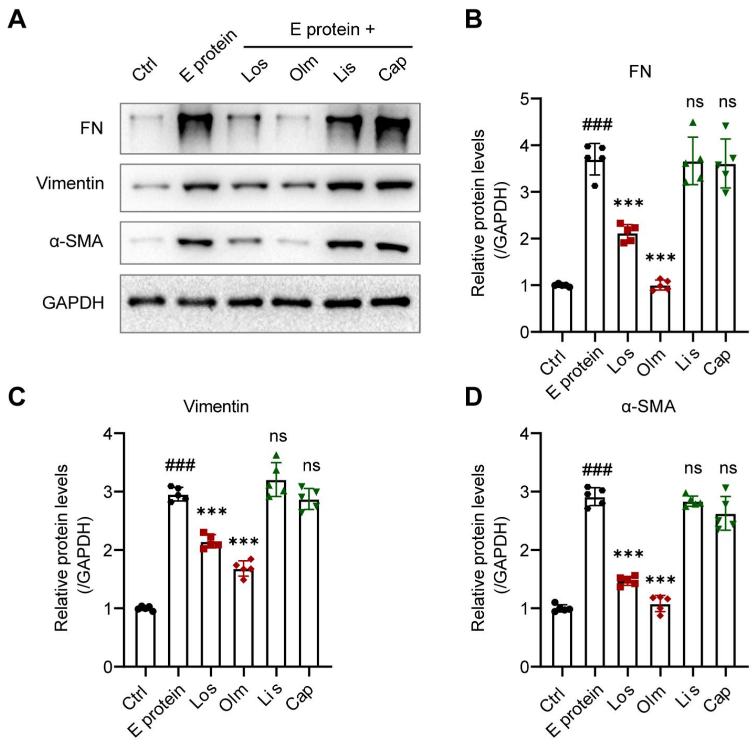

Supplementary Figure 1. Drug screening *in vitro*. (A) Representative ARBs ((losartan (Los) and olmesartan (Olm)) and ACEIs (lisinopril (Lis) and captopril (Cap)) inhibit E protein induced epithelial mesenchymal transformation (EMT) of HK-2 cells at 20 μM . (B-D) The relative quantitation of protein expression of fibronectin (FN), vimentin, and α-smooth muscle actin (α-SMA) (n=5). All the values are means ± SD; one-way ANOVA. ### $P < 0.001$ *vs.* the control group and ns $P > 0.05$, *** $P < 0.001$ *vs.* the E protein group.

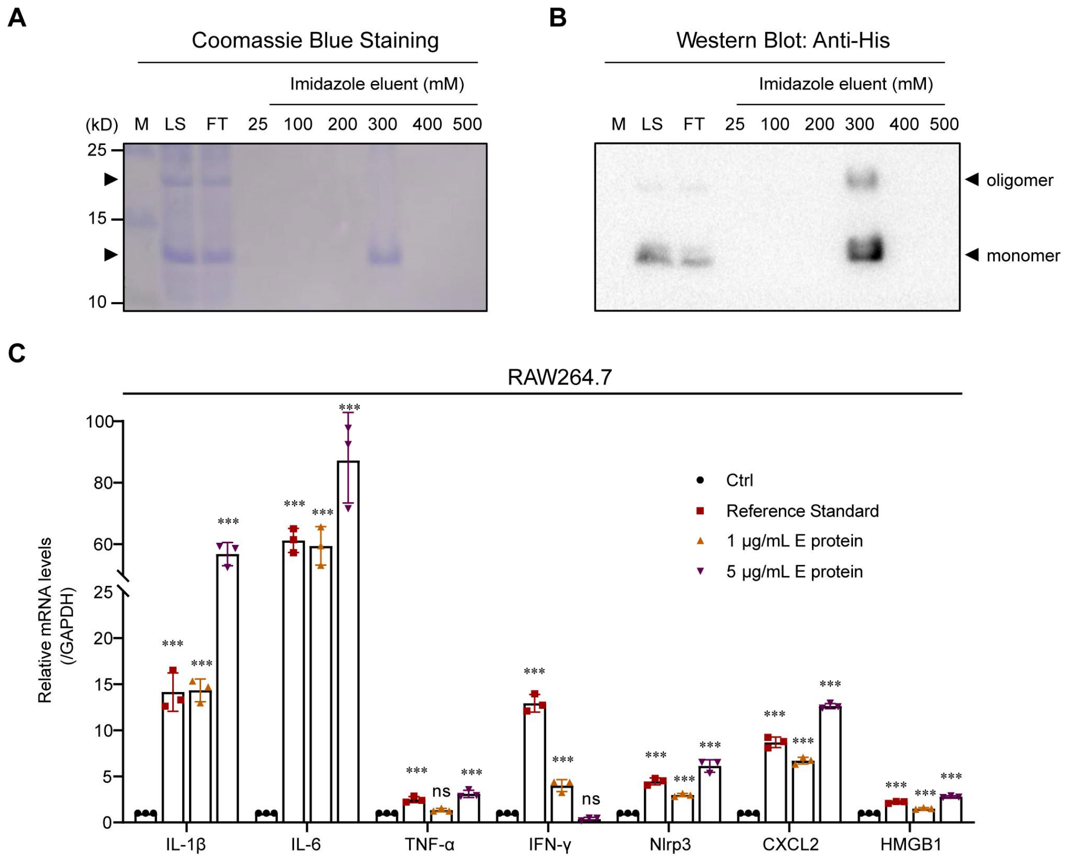

Supplementary Figure 2. Purification and validation of SARS-CoV-2 E proteins. (A) 15% SDS-PAGE gel with coomassie blue staining. (B) Western blot probed with anti-his-tag antibody. (C) qRT-PCR analysis of cytokines expression upon treatment with purified E protein and reference standard (E protein; Cat# RP01263, ABclonal Technology, Wuhan, China) (n=3). All the values are means ± SD; one-way ANOVA. *** $P < 0.001$ *vs.* the control group. M: maker; LS: lysate; FT: flowthrough fluid.

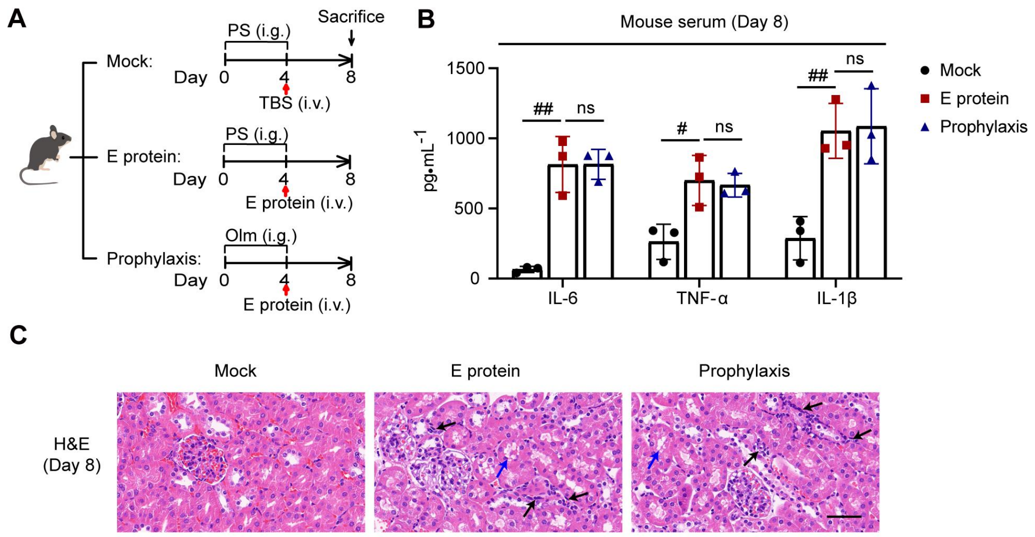

Supplementary Figure 3. Effect of prophylactic administration of olmesartan on E protein induced damage. (A) Schematic models show experimental design for animals treatments (n=3). (B) The level of IL-6, TNF-α and IL-1β in mouse serum (n=3). (C) Representative H&E images of kidney samples on day 8 of the experiment (blue arrow: vacuolar degeneration in renal tubular epithelium; black arrow: inflammatory cells infiltration in the renal interstitium; scale bar = 50 μm). All the values are means ± SD; one-way ANOVA. # $P < 0.05$, ## $P < 0.01$ *vs.* the mock group and ns $P > 0.05$ *vs.* the E protein group. (E protein group: 10 $mg \cdot kg^{-1}$ purified E protein; Prophylaxis group: 10 $mg \cdot kg^{-1} \cdot d^{-1}$ olmesartan)

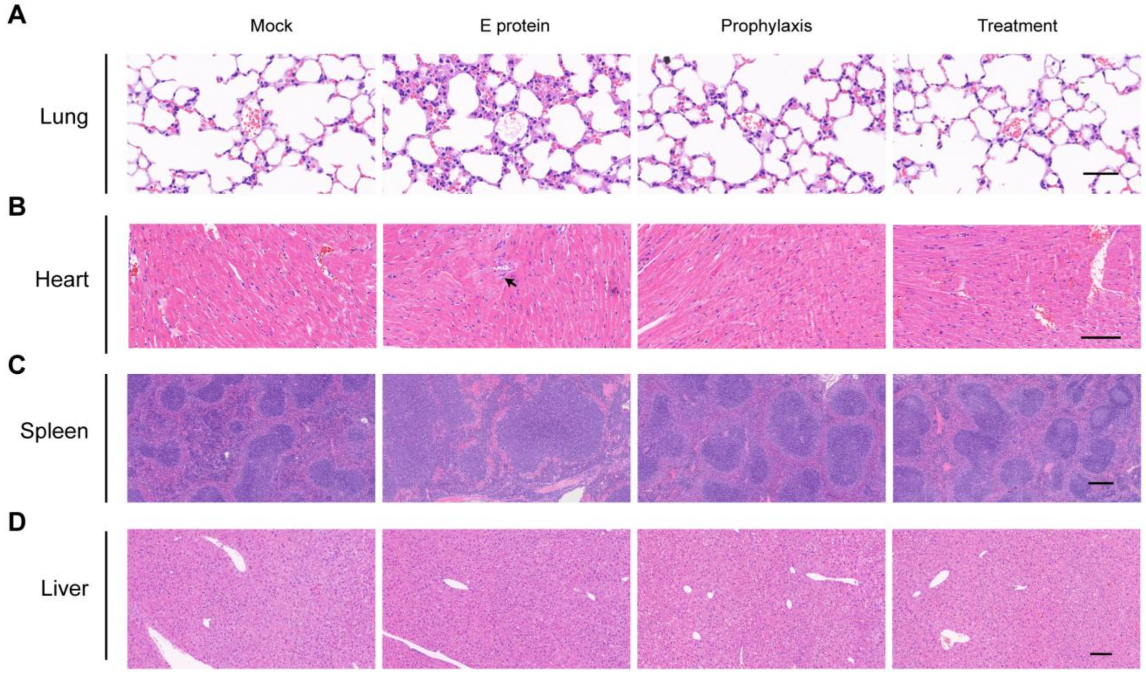

Supplementary Figure 4. Comparison of histopathological features. H&E staining of (A) lung, (B) heart (black arrow: fibrotic-like structures), (C) spleen and (D) liver. Scale bar = 100 μm.

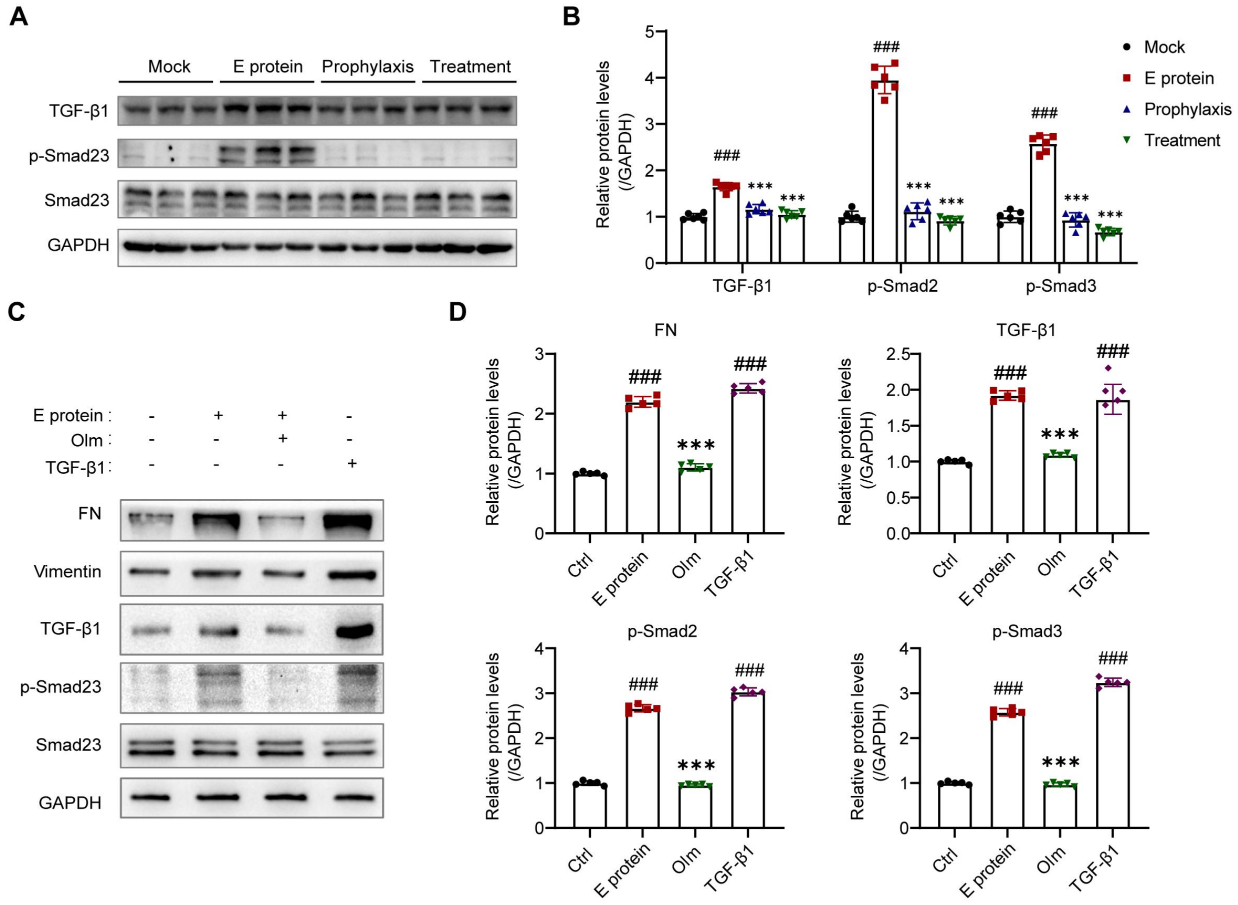

Supplementary Figure 5. Olmesartan inhibits the activation of TGF-β1/Smad2/3 pathway. (A) Activation of TGF-β1/Smad2/3 pathway in the kidneys from each group and (B) the relative quantitation (n=6). (C) Activation of TGF-β1/Smad2/3 pathway by E protein in HK-2 cells and the inhibition of Olmesartan and (D) the relative quantitation (n=5). All the values are means ± SD; one-way ANOVA. ### $P < 0.001$ *vs.* the mock or control group and ******* $P < 0.001$ *vs.* the E protein group.

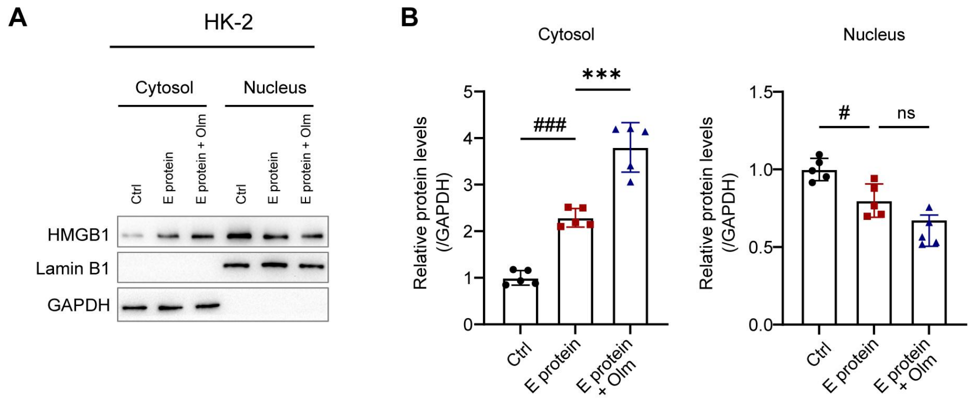

Supplementary Figure 6. Regulation of HMGB1 by Olmesartan in HK-2. (A) The distribution of HMGB1 in nucleus and cytoplasm in HK-2 cells and (B) the relative quantitation. All the values are means ± SD; one-way ANOVA. ns *P* > 0.05; # *P* < 0.05, ### *P* < 0.001 *vs.* the control group and *** *P* < 0.001 *vs.* the E protein group.

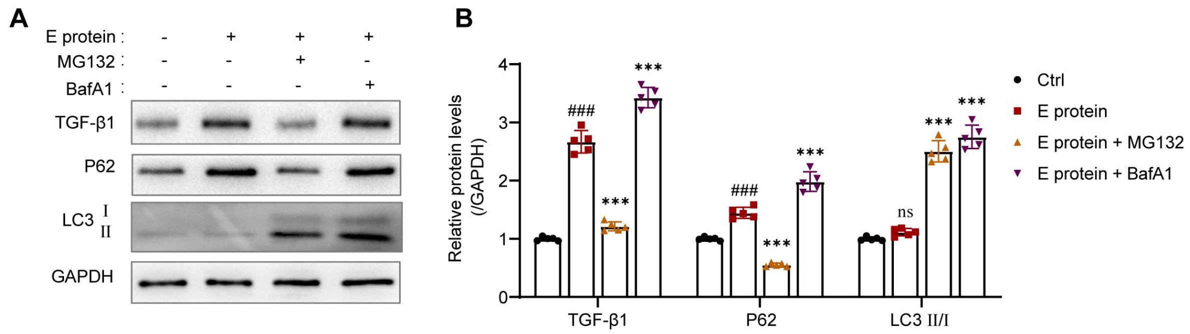

Supplementary Figure 7. TGF-β1 is degraded by autophagy. (A) TGF-β1 degradation pathway in HK-2 cells under E protein treatment and (B) the relative quantitation (n=5). All the values are means ± SD; one-way ANOVA. ns *P* > 0.05; ### *P* < 0.001 *vs.* the control group and *** *P* < 0.001 *vs.* the E protein group. MG132: proteasome inhibitor, also autophagy activator. BafA1: autophagy inhibitor.

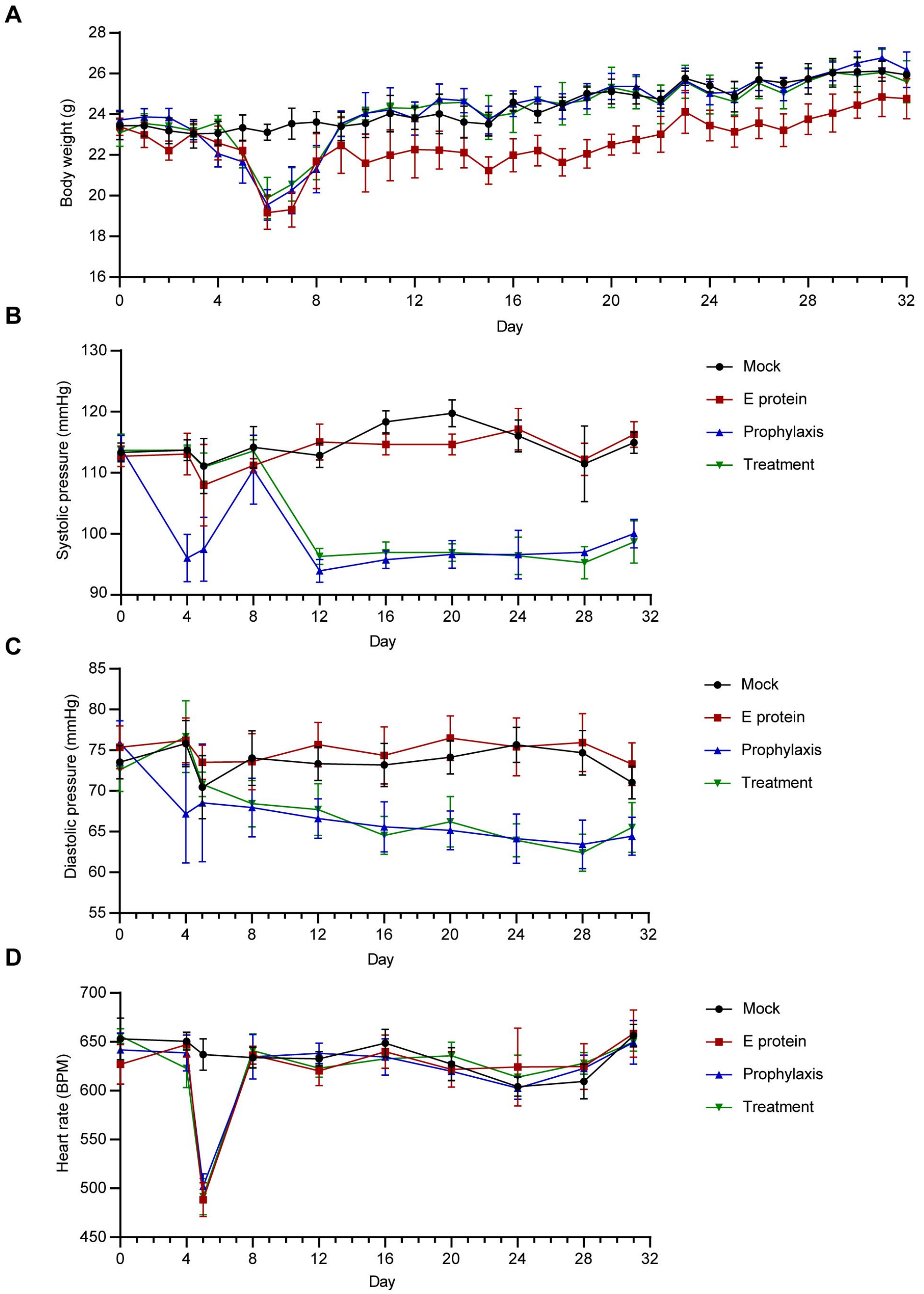

Supplementary Figure 8. Physiological indicators of model establishment and drug administration. Curve of changes in (A) body weight, (B) systolic pressure, (C) diastolic pressure and (D) heart rate during animal treatment (n=6).

Supplementary Table 1: List of Quantitative Real-time PCR (qRT-PCR) primers			
**Primer Name**	**Forward Sequence**	**Reverse Sequence**	**Species**
GAPDH	CATCACTGCCACCCAGAAGACTG	ATGCCAGTGAGCTTCCCGTTCAG	Mouse
Fibronectin	CCCTATCTCTGATACCGTTGTCC	TGCCGCAACTACTGTGATTCGG	Mouse
Collagen I	CCTCAGGGTATTGCTGGACAAC	CAGAAGGACCTTGTTTGCCAGG	Mouse
Vimentin	CGGAAAGTGGAATCCTTGCAGG	AGCAGTGAGGTCAGGCTTGGAA	Mouse
α-SMA	TGCTGACAGAGGCACCACTGAA	CAGTTGTACGTCCAGAGGCATAG	Mouse
TGF-β1	TGATACGCCTGAGTGGCTGTCT	CACAAGAGCAGTGAGCGCTGAA	Mouse
IL-1β	GATCCACACTCTCCAGCTGCA	CAACCAACAAGTGATATTCTCCATG	Mouse
IL-6	GACAAAGCCAGAGTCCTTCAGAGAG	CTAGGTTTGCCGAGTAGATCTC	Mouse
TNF-α	CATCTTCTCAAAATTCGAGTGACAA	TGGGAGTAGACAAGGTACAACCC	Mouse
IFN-γ	GAAAGACAATCAGGCCATCA	TTGCTGTTGCTGAAGAAGGT	Mouse
CXCL2	CAGACAGAAGTCATAGCCAC	TTCCAGGTCAGTTAGCCTTG	Mouse
HMGB1	AGGCTGACAAGGCTCGTTATGAAAG	GGGCGGTACTCAGAACAGAACAAG	Mouse
Nlrp3	CAGATTGCTGTGTGTGGGACTGA	AGCTCAGAACCAATGCGAGATCCT	Mouse
GAPDH	GTCTCCTCTGACTTCAACAGCG	ACCACCCTGTTGCTGTAGCCAA	Human
TGF-β1	TACCTGAACCCGTGTTGCTCTC	GTTGCTGAGGTATCGCCAGGAA	Human
